# Influence of Open Chain and Cyclic Structure of Peptidomimetics on Antibacterial Activity in *E*. *coli* Strains

**DOI:** 10.3390/molecules27113633

**Published:** 2022-06-06

**Authors:** Parul Sahrawat, Paweł Kowalczyk, Dominik Koszelewski, Mateusz Szymczak, Karol Kramkowski, Aleksandra Wypych, Ryszard Ostaszewski

**Affiliations:** 1 Institute of Organic Chemistry PAS, Kasprzaka 44/52, 01-224 Warsaw, Poland; parul@icho.edu.pl (P.S.); dominik.koszelewski@icho.edu.pl (D.K.); 2 Department of Animal Nutrition, The Kielanowski Institute of Animal Physiology and Nutrition, Polish Academy of Sciences, Instytucka 3, 05-110 Jabłonna, Poland; 3 Department of Molecular Virology, Faculty of Biology, Institute of Microbiology, University of Warsaw, Miecznikowa 1, 02-096 Warsaw, Poland; mszymczak@biol.uw.edu.pl; 4 Department of Physical Chemistry, Medical University of Bialystok, Kilińskiego 1 Str., 15-089 Białystok, Poland; kkramk@wp.pl; 5 Centre for Modern Interdisciplinary Technologies, Nicolaus Copernicus University in Torun, ul. Wileńska 4, 87-100 Toruń, Poland; wypych@umk.pl

**Keywords:** cyclic peptide, ugi multicomponent reaction, diketopiperazines, antimicrobial activity, minimal inhibitory concentration

## Abstract

An efficient method for the synthesis of functionalized peptidomimetics via multicomponent Ugi reaction has been developed. The application of trifluoroethanol (TFE) as a reaction medium provided desired products with good yields. Further, using the developed cyclisation reaction, the obtained peptidomimetics were transformed into the cyclic analogues (diketopiperazines, DKPs). The goal of the performed studies was to revised and compare whether the structure of the obtained structurally flexible acyclic peptidomimetics and their rigid cycling analogue DKPs affect antimicrobial activity. We studied the potential of synthesized peptidomimetics, both cyclic and acyclic, as antimicrobial drugs on model *E. coli* bacteria strains (k12, R2–R4). The biological assays reveal that DKPs hold more potential as antimicrobial drugs compared to open chain Ugi peptidomimetics. We believe that it can be due to the rigid cyclic structure of DKPs which promotes the membrane penetration in the cell of studied pathogens. The obtained data clearly indicate the high antibiotic potential of synthesized diketopiperazine derivatives over tested antibiotics.

## 1. Introduction

**Figure 1:**
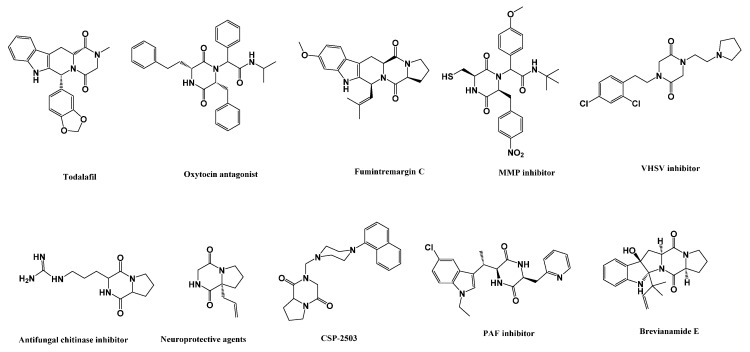
Biologically active DKPs.

Ugi reaction is a one pot multicomponent reaction for peptidomimetics synthesis. This transformation was already successfully engaged as a crucial step for diketopiperazines (DKPs) synthesis [1,2]. Diketopiperazine derivatives are known for their well-recognized biological activities. These small, cycling and conformationally rigid molecules have multiple functional groups which can interact with receptors and so show a broad spectrum of biological actions [3]. There are plentiful reports on bioactive compounds containing DKP moiety. This scaffold is quite important due to its interesting medicinal properties such as PDE5 inhibitors [4], oxytocin antagonists [5], cancer inhibitors [6], metalloproteinase inhibitors [7], antivirals [8], antibiotics [9], antibacterial [10], neuroprotective reagents [11], anxiolytic agents [12], anti-inflammatory inhibitors [13], bio herbicides [14] and natural products [15] (Figure 1).

This work is a continuation of earlier work reported by us, in which we paid attention to the structure–activity relationship of a library of peptidomimetics synthesized via Ugi reaction to check their antimicrobial potential against *E. coli* strains [16]. However, here, we are more interested in assessing the effect of cyclisation with respect to the open chain Ugi peptidomimetics. In order to study the influence of structure, particularly its rigidity, it is necessary to develop the method for the synthesis of these two groups of compounds. We have exploited the diversity of Ugi reaction using several aldehydes, isocyanides, amines and acrylic acid to make a library of compounds. Reported peptidomimetics are a class of alkylating agents which should aid its antimicrobial activity but our research has shown otherwise, which may be related to cell membrane permeability in pathogens. 2-chloropropionic acid derivatives are also proven to be biologically active. For example, piracetam derivatives are known as cognitive enhancers [17] and 3CL pro inhibitors [18]. Due to antimicrobial resistance, there is a need to find new potent antimicrobial drugs. There are very few reports on the antimicrobial activities of DKPs against *E. coli* [19]. The goal of the presented studies is to design a method for the synthesis of DKPs and to substantiate their antimicrobial potential against model *E. coli* strains.

## 2. Materials and Methods

### 2.1. Microorganisms and Media

*E. coli* K-12, R1–R4 strains were received from Prof. Jolanta Łukasiewicz at the Ludwik Hirszfeld Institute of Immunology and Experimental Therapy (Polish Academy of Sciences, Warsaw, Poland). Bacteria were cultivated in a tryptic soy broth (TSB; Sigma-Aldrich, Saint Louis, MI, USA) liquid medium and on agar plates containing TSB medium. N,N-Dimethylformamide (DMF) was purchased from Sigma Aldrich (CAS No. 68-12-2, Poznań, Poland), Lanes 1kb-ladder, and Quick Extend DNA ladder (New England Biolabs, Ipswich, MA, USA), with MIC and MBC tests as described in detail in the previous work [16,17,18,19,20,21,22] and analyzed by the Tukey test indicated by (*p* < 0.05): * *p* < 0.05, ** *p* < 0.1, *** *p* < 0.01.

### 2.2. Chemicals

Starting materials and all other reagents were purchased from Sigma-Aldrich. All solvents were of analytical grade and were used without prior distillation. Merck silica gel plates 60 F254 were used for TLC (thin layer chromatography) analysis. Crude reaction mixtures were purified using column chromatography on Merck silica gel 60/230–400 mesh, with an appropriate mixture of hexane and ethyl acetate as a solvent. THF was dried according to standard procedure. Nuclear magnetic resonance spectra (NMR) were performed on a Bruker Avance 400 and Varian 500MHz instrument. Chemical shifts are expressed in ppm and coupling constant (J) in Hz using TMS as an internal standard. High-resolution mass spectra were acquired on a Maldi SYNAPT G2-S HDMS (Waters) apparatus with a QqTOF analyser.

The bacterial tests used, and MIC and MBC were accurately described in detail in the previous work [20,21,22,23,24,25,26]. An MTT test to assess the metabolic activity of cells was performed on the basis of [27,28,29,30], with THLE-5b as the control and the caco-2 cell line derived from human adenocarcinoma.

### 2.3. General Procedure for Synthesis of Compounds Va-Vh

To the mixture of benzyl amine (0.25 mmol) in 2,2,2-trifluoroethanol (1 mL, 0.25 mmol) corresponding aldehyde was added and the reaction mixture was stirred for 20 min at room temperature followed by the addition of 2-chloropropionic acid (0.25 mmol). After 20 min, isocyanide (0.25 mmol) was added to the reaction mixture and was stirred continuously for 18 h at 50 °C. Then, the solvent was evaporated and the crude products were purified by column chromatography on silica gel using hexane/AcOEt as the eluent.

#### 2.3.1. N-benzyl-2-chloro-N-(2-((4-methoxybenzyl)amino)-2-oxo-1-phenylethyl) Propanamide (Va)

Colorless oil; (*dr*: 1:1); ^1^H NMR (400 MHz, CDCl_3_) δ 7.38 (dd, *J* = 6.5, 2.8 Hz, 2H), 7.29–7.15 (m, 8H), 7.05 (d, *J* = 7.3 Hz, 2H), 6.84–6.76 (m, 2H), 6.13 (s, 1H), 5.76 (s, 1H), 4.99 (d, *J* = 18.0 Hz, 1H), 4.61 (d, *J* = 18.0 Hz, 1H), 4.44–4.32 (m, 2H), 3.75 (s, 3H), 1.57 (d, *J* = 6.5 Hz, 3H); ^13^C NMR (100 MHz, CDCl_3_) δ 170.6, 168.7, 158.9, 136.9, 134.3, 130.0, 129.8, 129.0, 128.9, 128.8, 128.6, 127.3, 126.0, 125.8, 114.0, 64.1, 55.2, 50.2, 50.1, 43.2, 20.7. HR-MS (ESI): m/z calculated for C_26_H_27_ClN_2_O_3_ [M+Na]^+^ 449.1629, found 449.1632.

#### 2.3.2. N-benzyl-2-chloro-N-(2-((4-methoxybenzyl)amino)-1-(4-methoxyphenyl)-2-oxoethyl) Propanamide (Vb)

Pale yellow oil; (*dr*: 3:1); ^1^H NMR (400 MHz, CDCl_3_) δ 7.34–7.26 (m, 2H), 7.25–7.18 (m, 3H), 7.18–7.14 (m, 2H), 7.06 (d, *J* = 7.3 Hz, 2H), 6.83–6.79 (m, 2H), 6.78–6.74 (m, 2H), 6.03–5.91 (m, 1H), 5.62 (s, 1H), 5.56 (d, *J* = 14.6 Hz, 1H, *minor*), 5.07 (t, *J* = 3.6 Hz, 2H, *minor*), 4.96 (d, *J* = 18.0 Hz, 1H, *major*), 4.56 (d, *J* = 18.0 Hz, 1H, *major*), 4.40 (t, *J* = 5.1 Hz, 2H, *major*), 3.77 (s, 3H), 3.73 (s, 3H), 3.57 (d, *J* = 14.6 Hz, 1H, *minor*), 1.64 (d, *J* = 7.2 Hz, 1H, *minor*), 1.59 (d, *J* = 6.4 Hz, 3H, *major*); ^13^C NMR (100 MHz, CDCl_3_) δ 168.9, 159.9, 159.80, 136.9, 131.1, 128.9, 128.7, 128.6, 128.1, 127.3, 125.8, 114.8, 114.3, 114.1, 63.7, 55.2, 50.2, 49.9, 43.2, 20.7. HR-MS (ESI): m/z calculated for C_27_H_29_ClN_2_O_4_ [M+Na]^+^ 503.1709, found 503.1714.

#### 2.3.3. N-benzyl-2-chloro-N-(2-(cyclohexylamino)-2-oxo-1-phenylethyl) Propanamide (Vc)

Colorless oil; (*dr*: 3:2); ^1^H NMR (500 MHz, CDCl_3_) δ 7.39–7.30 (m, 3H), 7.30–7.23 (m, 6H), 7.22–7.18 (m, 2H), 7.12 (d, *J* = 7.5 Hz, 2H), 7.03 (d, *J* = 7.5 Hz, 1H), 6.04 (s, 1H, *major*), 5.79 (d, *J* = 8.0 Hz, 1H, *major*), 5.70 (s, 1H, *minor*), 5.57 (d, *J* = 7.9 Hz, 1H, *minor*), 4.98 (d, *J* = 18.0 Hz, 1H, *minor*), 4.66 (q, *J* = 17.6 Hz, 2H), 4.56 (d, *J* = 18.1 Hz, 1H*, minor*), 4.47 (q, *J* = 6.6 Hz, 1H, *major*), 4.38 (q, *J* = 6.6 Hz, 1H, *major*), 3.80 (ddtt, *J* = 22.7, 11.2, 7.8, 4.0 Hz, 2H), 1.89 (dd, *J* = 11.5, 5.5 Hz, 3H), 1.75–1.60 (m, 5H), 1.59 (dd, *J* = 6.5, 2.3 Hz, 5H), 1.38–1.23 (m, 4H), 1.15–1.02 (m, 4H); ^13^C NMR (126 MHz, CDCl_3_) δ 171.5, 170.5, 167.8, 167.7, 137.4, 136.9, 134.8, 134.4, 129.7, 129.1, 128.8, 128.7, 128.6, 128.6, 128.5, 127.9, 127.3, 127.2, 126.0, 125.9, 125.7, 64.1, 62.7, 50.8, 50.2, 50.0, 49.6, 48.5, 34.6, 32.8, 32.7, 32.7, 32.6, 31.5, 29.0, 25.4, 25.4, 25.2, 24.7, 24.6, 21.0, 20.7, 20.6. HR-MS (ESI): m/z calculated for C_24_H_29_ClN_2_O_2_ [M+Na]^+^ 435.1812, found 435.1815.

#### 2.3.4. N-benzyl-2-chloro-N-(2-((4-methoxybenzyl)amino)-2-oxo-1-(p-tolyl)ethyl) Propanamide (Vd)

White semi-solid; (*dr*: 3:2); ^1^H NMR (400 MHz, CDCl_3_) δ 7.28–7.17 (m, 9H), 7.13 (dt, *J* = 8.3, 4.6 Hz, 5H), 7.10–6.97 (m, 6H), 6.86–6.77 (m, 4H), 6.32 (d, *J* = 7.1 Hz, 1H, *major*), 6.03 (s, 1H, *minor*), 5.99 (d, *J* = 5.9 Hz, 1H, *minor*), 5.61 (s, 1H, *major*), 4.97 (d, *J* = 18.0 Hz, 1H), 4.64 (d, *J* = 11.7 Hz, 1H), 4.55 (d, *J* = 18.1 Hz, 1H), 4.38 (ddd, *J* = 13.2, 7.3, 4.0 Hz, 4H), 3.77 (d, *J* = 2.1 Hz, 6H), 2.29 (s, 3H, *minor*), 2.26 (s, 3H, *major*), 1.57 (dd, *J* = 9.0, 6.5 Hz, 5H); ^13^C NMR (100 MHz, CDCl_3_) δ 170.5, 168.8, 158.9, 138.5, 136.9, 129.1, 128.9, 128.6, 127.2, 125.8, 114.0, 114.1, 64.2, 62.8, 55.2, 50.8, 50.2, 50.1, 43.2, 21.1, 20.7. HR-MS (ESI): m/z calculated for C_27_H_29_ClN_2_O_3_ [M+Na]^+^ 487.1771, found 487.1764.

#### 2.3.5. N-benzyl-2-chloro-N-(2-(cyclohexylamino)-2-oxo-1-(p-tolyl)ethyl) Propanamide (**Ve**)

Colorless oil; (*dr*: 3:2)**;** ^1^H NMR (400 MHz, CDCl_3_) δ 7.32–7.09 (m, 10H), 7.05 (q, *J* = 8.3 Hz, 5H), 6.02 (s, 1H, *minor*), 5.95 (d, J = 8.1 Hz, 1H, *minor*), 5.64 (d, *J* = 8.4 Hz, 2H, *major*), 4.94 (d, *J* = 18.0 Hz, 1H, *major*), 4.68 (d, *J* = 17.7 Hz, 2H, *minor*), 4.54 (d, *J* = 17.7 Hz, 1H, *major*), 4.44 (q, *J* = 6.4 Hz, 1H, *minor*), 4.36 (q, *J* = 6.5 Hz, 1H, *major*), 3.88–3.68 (m, 2H), 2.29 (s, 3H, *minor*), 2.26 (s, 3H, *major*), 1.96–1.81 (m, 3H), 1.69–1.59 (m, 5H), 1.55 (d, *J* = 6.5 Hz, 6H), 1.38–1.22 (m, 5H), 1.09 (dqd, *J* = 17.1, 11.7, 11.1, 4.1 Hz, 5H); ^13^C NMR (100 MHz, CDCl_3_) δ 175.6, 174.9, 169.8, 169.5, 168.1, 137.7, 129.4, 128.6, 128.5, 128.1, 127.2, 127.1, 126.1, 126.0, 56.3, 48.3, 48.0, 43.1, 42.7, 37.1, 32.8, 32.7, 32.6, 32.5, 29.4, 25.9, 25.5, 25.1, 24.7, 24.6, 24.6, 22.7, 22.6, 22.4, 22.4, 22.3. HR-MS (ESI): m/z calculated for C_25_H_31_ClN_2_O_2_ [M+Na]^+^ 449.1970, found 449.1972.

#### 2.3.6. N-benzyl-2-chloro-N-(2-(cyclohexylamino)-1-(2,4-dinitrophenyl)-2-oxoethyl) Propanamide (**If**)

Yellow oil; (*dr*: 3:2); ^1^H NMR (400 MHz, CDCl_3_) δ 8.79 (d, *J* = 2.4 Hz, 1H, *major*), 8.67–8.60 (m, 1H, *minor*), 8.43 (dd, *J* = 8.6, 2.4 Hz, 1H, *major*), 8.35–8.28 (m, 1H, *minor*), 7.79 (d, *J* = 8.7 Hz, 1H, *minor*), 7.70 (d, *J* = 8.7 Hz, 1H, *major*), 7.30 (dd, *J* = 14.5, 6.9 Hz, 4H), 7.24 (s, 2H), 7.16 (d, *J* = 7.4 Hz, 3H), 7.09 (d, *J* = 6.7 Hz, 2H), 6.63 (s, 1H, major), 6.37 (s, 1H, minor), 6.28 (d, *J* = 7.9 Hz, 1H, *major*), 5.87 (d, *J* = 8.0 Hz, 1H, *minor*), 5.07 (d, *J* = 17.0 Hz, 2H, *major*), 4.86 (d, *J* = 17.7 Hz, 1H, *minor*), 4.61 (dt, *J* = 10.9, 5.5 Hz, 2H), 3.74–3.51 (m, 1H, *minor*), 3.31–3.17 (m, 1H*, major*), 1.72 (d, *J* = 6.5 Hz, 4H), 1.66 (d, *J* = 6.4 Hz, 4H), 1.62–1.55 (m, 4H), 1.54–1.44 (m, 4H), 1.29–1.16 (m, 5H), 1.12–0.93 (m, 7H); ^13^C NMR (126 MHz, CDCl_3_) δ 170.7, 170.2, 164.5, 149.7, 149.2, 147.2, 138.6, 136.0, 135.2, 131.7, 129.7, 129.3, 129.1, 128.4, 128.1, 127.2, 126.8, 126.5, 125.9, 120.4, 120.2, 60.7, 60.0, 51.5, 50.0, 49.8, 49.2, 48.9, 32.5, 31.7, 25.3, 25.3, 24.4, 20.7, 20.3. HR-MS (ESI): m/z calculated for C_24_H_27_ClN_4_O_6_ [M-H]^+^ 501.1537, found 501.1541.

#### 2.3.7. N-benzyl-2-chloro-N-(2-(cyclohexylamino)-1-(4-(dimethylamino)phenyl)-2-oxoethyl) Propanamide (Vg)

Pale yellow oil; (*dr*: 3:2); ^1^H NMR (400 MHz, CDCl_3_) δ 7.31 (q, *J* = 2.5, 1.6 Hz, 1H), 7.19 (dt, *J* = 18.3, 7.1 Hz, 12H), 7.08 (d, *J* = 7.5 Hz, 2H), 6.59 (dd, *J* = 8.5, 4.0 Hz, 4H), 5.92 (s, 1H), 5.67 (d, *J* = 8.1 Hz, 1H), 5.54 (d, *J* = 8.4 Hz, 2H), 4.92 (d, *J* = 18.0 Hz, 1H), 4.66–4.48 (m, 3H), 4.44 (d, *J* = 6.5 Hz, 1H), 4.34 (q, *J* = 6.4 Hz, 1H), 3.78 (ddtd, *J* = 20.1, 16.5, 8.6, 8.2, 4.3 Hz, 2H), 2.90 (d, *J* = 4.6 Hz, 12H), 1.99–1.82 (m, 5H), 1.56 (t, *J* = 6.6 Hz, 7H), 1.38–1.24 (m, 7H), 1.15–1.00 (m, 7H); ^13^C NMR (100 MHz, CDCl_3_) δ 170.4, 168.4, 150.5, 137.5, 130.7, 130.4, 129.6, 128.6, 128.5, 128.3, 127.1, 127.1, 126.2, 125.8, 112.3, 112.3, 64.0, 62.8, 51.1, 50.3, 49.7, 49.2, 48.5, 40.2, 32.7, 26.9, 25.5, 24.8, 24.7, 21.1, 20.7. HR-MS (ESI): m/z calculated for C_26_H_34_ClN_3_O_2_ [M+Na]^+^ 478.2239, found 478.2237.

#### 2.3.8. N-benzyl-2-chloro-N-(2-(cyclohexylamino)-1-(4-nitrophenyl)-2-oxoethyl) Propanamide (Vh)

Colorless oil; (*dr*: 3:2); ^1^H NMR (400 MHz, CDCl_3_) δ 8.24–8.19 (m, 1H, *minor*), 8.11–8.07 (m, 1H, *major*), 8.02 (d, *J* = 8.7 Hz, 2H), 7.50 (dd, *J* = 16.3, 8.7 Hz, 4H), 7.31–7.29 (m, 2H), 7.16 (h, *J* = 4.4, 3.6 Hz, 4H), 7.10 (d, *J* = 7.4 Hz, 2H), 6.95–6.90 (m, 2H), 6.40 (d, *J* = 7.9 Hz, 1H, *major*), 6.06 (s, 1H, *minor*), 6.01 (d, *J* = 8.1 Hz, 1H, *minor*), 5.88 (s, 1H, *major*), 5.40 (d, *J* = 14.7 Hz, 1H, *minor*), 5.00 (d, *J* = 17.8 Hz, 1H, *major*), 4.60 (d, *J* = 17.8 Hz, 1H, *major*), 4.50 (q, *J* = 5.6, 4.7 Hz, 1H, *minor*), 4.45 (dd, *J* = 8.3, 4.7 Hz, 1H, *major*), 3.93 (d, *J* = 14.7 Hz, 1H, *minor*), 3.84–3.64 (m, 2H), 1.92–1.82 (m, 2H), 1.67–1.54 (m, 14H), 1.34–1.25 (m, 4H), 1.12 (q, *J* = 12.0, 10.1 Hz, 6H); ^13^C NMR (126 MHz, CDCl_3_) δ 171.1, 170.7, 166.6, 164.5, 148.4, 147.7, 142.3, 141.5, 138.8, 135.9, 130.5, 129.4, 129.2, 128.9, 128.8, 128.7, 128.6, 128.4, 127.9, 127.8, 127.1, 126.0, 125.7, 124.6, 124.5, 123.7, 123.6, 63.1, 61.9, 61.5, 60.3, 50.5, 50.3, 50.1, 48.7, 48.6, 32.7, 32.6, 31.1, 25.3, 24.6, 24.0, 21.0, 20.6, 20.5, 16.6, 14.2. HR-MS (ESI): m/z calculated for C_24_H_28_ClN_3_O_4_ [M+Na]^+^ 480.1668, found 480.1666.

### 2.4. General Procedure for Synthesis of Compounds VIa-VIh

Compound **V** (0.25 mmol) obtained in step 1 was dissolved in THF (3 mL) and cooled to 0 °C in an ice bath. After 5 min, sodium hydride (NaH 3 eq.) was added to it portion wise and the reaction mixture was refluxed for 3 hrs. The solvent was evaporated and the crude mixture was purified by column chromatography on silica gel using hexane/AcOEt as the eluent.

#### 2.4.1. 1-benzyl-4-(4-methoxybenzyl)-3-methyl-6-phenylpiperazine-2,5-dione (VIa)

Colorless oil; (*dr*: 99.9:0.1); ^1^H NMR (500 MHz, CDCl_3_) δ 7.42 (d, *J* = 3.8 Hz, 5H), 7.22–7.14 (m, 5H), 6.92 (d, *J* = 8.5 Hz, 2H), 6.81 (d, *J* = 8.6 Hz, 2H), 5.85 (s, 1H), 4.67 (d, *J* = 15.4 Hz, 1H), 4.25 (dd, *J* = 14.3, 6.1 Hz, 1H), 4.03 (dd, *J* = 14.3, 6.1 Hz, 1H), 3.83 (s, 3H), 3.79 (dd, *J* = 8.4, 6.5 Hz, 1H), 3.64 (d, *J* = 15.4 Hz, 1H), 1.33 (d, *J* = 7.5 Hz, 3H); ^13^C NMR (126 MHz, CDCl_3_) δ 172.1, 168.4, 159.1, 136.3, 135.9, 129.3, 129.1, 129.0, 128.7, 128.5, 128.1, 127.4, 114.0, 57.5, 55.3, 45.0, 42.9, 30.3, 29.7, 11.1. HR-MS (ESI): m/z calculated for C_26_H_26_N_2_O_3_ [M+Na]^+^ 437.1792, found 437.1796.

#### 2.4.2. 1-benzyl-4-(4-methoxybenzyl)-6-(4-methoxyphenyl)-3-methylpiperazine-2,5-dione (VIb)

Pale yellow oil; (*dr*: 95:5); ^1^H NMR (500 MHz, CDCl_3_) δ 7.32–7.29 (m, 2H), 7.20–7.12 (m, 5H), 6.92–6.88 (m, 4H), 6.81–6.76 (m, 2H), 5.83 (s, 1H), 4.64 (d, *J* = 15.3 Hz, 1H), 4.21 (dd, *J* = 14.2, 6.1 Hz, 1H), 4.00 (dd, *J* = 14.3, 6.1 Hz, 1H), 3.73 (q, *J* = 7.5 Hz, 1H), 3.61 (d, *J* = 15.3 Hz, 1H), 1.29 (d, *J* = 7.5 Hz, 3H); ^13^C NMR (126 MHz, CDCl_3_) δ 172.2, 168.7, 159.7, 159.1, 136.4, 129.5, 129.3, 129.1, 128.8, 128.5, 128.1, 127.7, 114.3, 113.9, 71.3, 57.4, 55.4, 55.3, 44.8, 42.9, 11.1. HR-MS (ESI): m/z calculated for C_27_H_28_N_2_O_4_ [M+Na]^+^ 467.1946, found 467.1947.

#### 2.4.3. 1-benzyl-4-cyclohexyl-3-methyl-6-phenylpiperazine-2,5-dione (VIc)

Colorless Oil; (*dr*: 85:15); ^1^H NMR (500 MHz, CDCl_3_) δ 7.44 (d, *J* = 4.2 Hz, 4H), 7.42–7.31 (m, 6H), 7.27 (dd, *J* = 8.4, 2.2 Hz, 3H), 5.56 (d, *J* = 8.3 Hz, 1H, *major*), 5.29 (d, *J* = 8.3 Hz, 1H, *minor*), 4.86 (d, *J* = 15.8 Hz, 1H, *minor*), 4.74 (d, *J* = 15.4 Hz, 1H, *major*), 4.15 (d, *J* = 15.8 Hz, 1H, *minor*), 4.11–4.02 (m, 1H, *minor*), 3.81 (q, *J* = 7.5 Hz, 1H), 3.67 (dtd, *J* = 11.3, 7.7, 4.0 Hz, 1H, *major*), 3.51 (d, *J* = 15.4 Hz, 1H, *major*), 1.73–1.63 (m, 1H), 1.56 (dd, *J* = 10.9, 6.7 Hz, 5H), 1.37 (d, *J* = 7.5 Hz, 3H, *major*), 1.31–1.19 (m, 4H), 0.96 (ddd, *J* = 17.6, 13.5, 10.5 Hz, 1H), 0.85 (d, *J* = 7.6 Hz, 3H, *minor*), 0.62 (qd, *J* = 12.2, 3.5 Hz, 1H), 0.49–0.39 (m, 1H); ^13^C NMR (126 MHz, CDCl_3_) δ 172.5, 167.6, 136.8, 136.3, 136.1, 134.3, 129.4, 128.9, 128.8, 128.8, 128.7, 128.6, 128.5, 128.3, 128.2, 127.9, 127.7, 127.5, 125.9, 71.7, 71.5, 57.4, 54.8, 48.5, 48.0, 45.1, 44.8, 32.6, 32.5, 32.4, 32.1, 29.6, 25.3, 25.2, 24.7, 24.6, 11.1, 10.2. HR-MS (ESI): m/z calculated for C_24_H_28_N_2_O_2_ [M+Na]^+^ 399.2048, found 399.2073.

#### 2.4.4. 1-benzyl-4-(4-methoxybenzyl)-3-methyl-6-(p-tolyl)piperazine-2,5-dione (VId)

White sticky compound; (*dr*: 3:2); ^1^H NMR (500 MHz, CDCl_3_) δ 7.27 (dd, *J* = 4.8, 2.6 Hz, 6H), 7.25–7.19 (m, 4H), 7.20–7.15 (m, 6H), 7.14 (dd, *J* = 6.6, 3.2 Hz, 2H), 7.12–7.09 (m, 1H), 6.92–6.87 (m, 4H), 6.81–6.75 (m, 4H), 5.82 (d, *J* = 6.3 Hz, 1H, *major*), 5.61 (s, 1H, *minor*), 4.80 (d, *J* = 15.7 Hz, 1H, *minor*), 4.64 (d, *J* = 15.4 Hz, 1H, *major*), 4.23 (d, *J* = 6.1 Hz, 1H, *minor*), 4.20 (d, *J* = 6.1 Hz, 1H, *minor*), 4.17–4.14 (m, 1H, *major*), 4.13 (d, *J* = 5.2 Hz, 1H), 4.04–4.01 (m, 1H, *minor*), 4.01–3.99 (m, 1H), 3.81 (s, 3H, *major*), 3.78 (s, 3H, *minor*), 3.75 (d, *J* = 7.5 Hz, 1H), 3.63 (d, *J* = 15.4 Hz, 1H, *major*), 2.36 (s, 3H, *major*), 2.35 (s, 3H, *minor*), 1.30 (d, *J* = 7.4 Hz, 3H, *major*), 1.21 (d, *J* = 6.2 Hz, 3H, *minor*); ^13^C NMR (126 MHz, CDCl_3_) δ 172.3, 172.2, 169.8, 168.6, 159.0, 138.6, 138.5, 136.4, 136.0, 132.8, 131.1, 129.6, 129.5, 129.5, 129.3, 129.1, 128.8, 128.5, 128.1, 128.0, 127.8, 127.5, 127.3, 114.0, 113.9, 71.52, 71.4, 64.3, 57.3, 55.3, 55.2, 54.3, 45.1, 44.9, 43.3, 42.9, 25.3, 21.0, 11.1, 10.3. HR-MS (ESI): m/z calculated for C_27_H_28_N_2_O_3_ [M+Na]^+^ 451.1997, found 451.1998.

#### 2.4.5. 1-benzyl-4-cyclohexyl-3-methyl-6-(p-tolyl)piperazine-2,5-dione (VIe)

Colorless Oil; (*dr*: 85:15); ^1^H NMR (500 MHz, CDCl_3_) δ 7.34 (q, *J* = 7.8, 7.0 Hz, 3H), 7.31–7.23 (m, 4H), 7.20 (d, *J* = 8.0 Hz, 2H), 5.52 (d, *J* = 8.3 Hz, 1H, *major*), 5.28 (d, *J* = 7.9 Hz, 1H, *minor*), 4.80 (d, *J* = 15.7 Hz, 1H, *minor*), 4.69 (d, *J* = 15.4 Hz, 1H, *major*), 4.11 (d, *J* = 15.8 Hz, 1H, *minor*), 4.05 (q, *J* = 7.6 Hz, 1H, *minor*), 3.75 (q, *J* = 7.5 Hz, 1H, *major*), 3.64 (dtd, *J* = 11.7, 8.0, 4.0 Hz, 1H), 3.49 (d, *J* = 15.4 Hz, 1H, *major*), 2.36 (s, 3H), 1.68–1.58 (m, 1H), 1.52 (d, *J* = 14.2 Hz, 4H), 1.33 (d, *J* = 7.5 Hz, 3H), 1.29–1.15 (m, 5H); ^13^C NMR (126 MHz, CDCl_3_) δ 172.58, 169.13, 167.74, 138.57, 138.37, 136.89, 136.43, 132.88, 131.12, 129.63, 129.43, 129.33, 128.94, 128.86, 128.25, 128.17, 127.87, 127.64, 127.38, 71.37, 57.21, 54.61, 48.52, 48.01, 47.89, 45.02, 44.71, 32.63, 32.59, 32.49, 32.11, 31.52, 29.63, 26.87, 25.24, 24.76, 24.74, 24.64, 21.01, 20.99, 14.04, 11.11, 10.19. HR-MS (ESI): m/z calculated for C_25_H_30_N_2_O_2_ [M-H]^+^ 389.2225, found 389.2229.

#### 2.4.6. 1-benzyl-4-cyclohexyl-6-(2,4-dinitrophenyl)-3-methylpiperazine-2,5-dione (VIf)

Yellow oil; (*dr*: 85:15)**;** ^1^H NMR (500 MHz, CDCl_3_) δ 8.91 (d, *J* = 2.4 Hz, 1H), 8.21 (dd, *J* = 8.5, 2.4 Hz, 1H), 7.37 (d, *J* = 8.6 Hz, 1H), 7.33 (q, *J* = 2.9 Hz, 5H), 5.54 (d, *J* = 7.6 Hz, 1H), 4.55 (d, *J* = 15.1 Hz, 1H), 4.26 (d, *J* = 15.1 Hz, 1H), 4.10–4.07 (m, 1H), 3.51 (dtdt, *J* = 14.3, 10.4, 6.4, 3.4 Hz, 1H), 1.71–1.50 (m, 10H), 1.45–1.41 (m, 1H), 1.40–1.19 (m, 7H), 0.91 (d, *J* = 7.4 Hz, 3H); ^13^C NMR (126 MHz, CDCl_3_) δ 171.0, 165.5, 149.0, 147.7, 138.6, 135.3, 131.28, 129.3, 129.2, 129.0, 128.8, 128.3, 126.9, 120.6, 72.7, 64.3, 56.5, 49.6, 49.2, 46.3, 33.9, 32.8, 32.4, 30.6, 29.7, 25.6, 25.3, 24.9, 24.8, 24.7, 17.6, 14.1, 13.6, 11.2. HR-MS (ESI): m/z calculated for C_24_H_26_N_4_O_6_ [M+Na]^+^ 489.1793, found 489.1795.

#### 2.4.7. 1-benzyl-4-cyclohexyl-6-(4-(dimethylamino)phenyl)-3-methylpiperazine-2,5-dione (VIg)

Yellow oil; (*dr*: 85:15); ^1^H NMR (500 MHz, CDCl_3_) δ 7.38–7.33 (m, 3H), 7.30–7.25 (m, 5H), 6.75–6.72 (m, 2H), 5.54 (d, *J* = 8.4 Hz, 1H), 4.69 (d, *J* = 15.3 Hz, 1H), 3.75 (q, *J* = 7.5 Hz, 1H), 3.67 (tdq, *J* = 11.6, 7.9, 3.8 Hz, 1H), 3.56 (d, *J* = 15.3 Hz, 1H), 3.00 (s, 5H), 1.61–1.49 (m, 5H), 1.35 (d, *J* = 7.5 Hz, 3H), 1.28–1.18 (m, 3H), 1.00–0.92 (m, 1H), 0.90–0.85 (m, 1H); ^13^C NMR (126 MHz, CDCl_3_) δ 172.9, 172.8, 169.6, 168.1, 150.4, 150.1, 137.1, 136.6, 129.2, 128.8, 128.7, 128.4, 128.2, 128.1, 127.7, 122.6, 120.5, 112.3, 112.0, 76.7, 71.5, 71.4, 60.3, 56.8, 54.5, 48.4, 47.9, 47.8, 44.8, 44.6, 40.2, 40.2, 32.6, 32.6, 32.5, 32.1, 25.3, 25.2, 24.7, 24.7, 24.6, 20.9, 14.1, 11.1, 10.2. HR-MS (ESI): m/z calculated for C_24_H_33_N_3_O_2_ [M+H]^+^ 442.2474 found 442.2470.

#### 2.4.8. 1-benzyl-4-cyclohexyl-3-methyl-6-(4-nitrophenyl)piperazine-2,5-dione (VIh)

Colorless oil; (*dr*: 1:1); ^1^H NMR (500 MHz, CDCl_3_) δ 8.28–8.22 (m, 4H), 7.69–7.65 (m, 2H), 7.58–7.54 (m, 2H), 7.42–7.33 (m, 9H), 7.28–7.26 (m, 2H), 5.62 (d, *J* = 8.3 Hz, 1H), 5.36 (d, *J* = 7.9 Hz, 1H), 4.90 (d, *J* = 15.6 Hz, 1H), 4.83 (d, *J* = 15.4 Hz, 1H), 4.16–4.10 (m, 1H), 4.02 (q, *J* = 7.6 Hz, 1H), 3.74 (q, *J* = 7.5 Hz, 1H), 3.63 (ddtd, *J* = 26.1, 11.3, 7.8, 3.9 Hz, 2H), 3.47 (d, *J* = 15.3 Hz, 1H), 1.74–1.64 (m, 3H), 1.64–1.50 (m, 9H), 1.38 (d, *J* = 7.5 Hz, 3H), 1.31–1.20 (m, 6H), 1.04–0.91 (m, 2H), 0.87 (d, *J* = 7.6 Hz, 3H); ^13^C NMR (126 MHz, CDCl_3_) δ 171.6, 171.5, 167.9, 166.5, 147.8, 143.5, 141.7, 136.2, 135.9, 129.6, 129.3, 128.9, 128.8, 128.7, 128.6, 128.5, 128.4, 123.9, 123.6, 76.7, 71.3, 70.7, 60.3, 59.1, 56.3, 48.7, 48.3, 45.4, 45.1, 32.6, 32.4, 32.1, 25.1, 24.7, 24.7, 24.6, 20.9, 14.1, 11.1, 10.2. HR-MS (ESI): m/z calculated for C_24_H_27_N_3_O_4_ [M+H]^+^ 444.1904 found 444.1899.

## 3. Results & Discussion

### 3.1. Chemistry

**Scheme 1:**
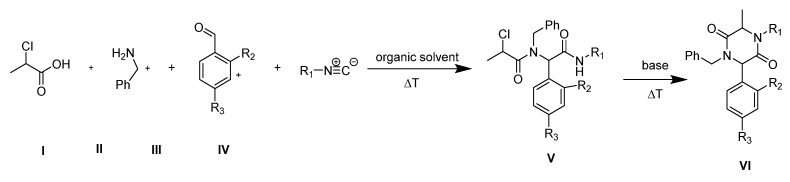
Proposed two step synthesis of DKPs.

**Scheme 2:**
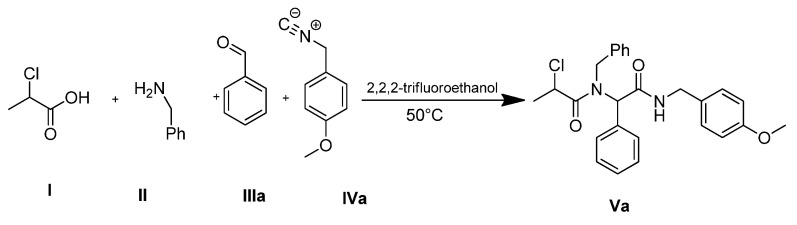
Model Ugi reaction.

**Figure 2:**
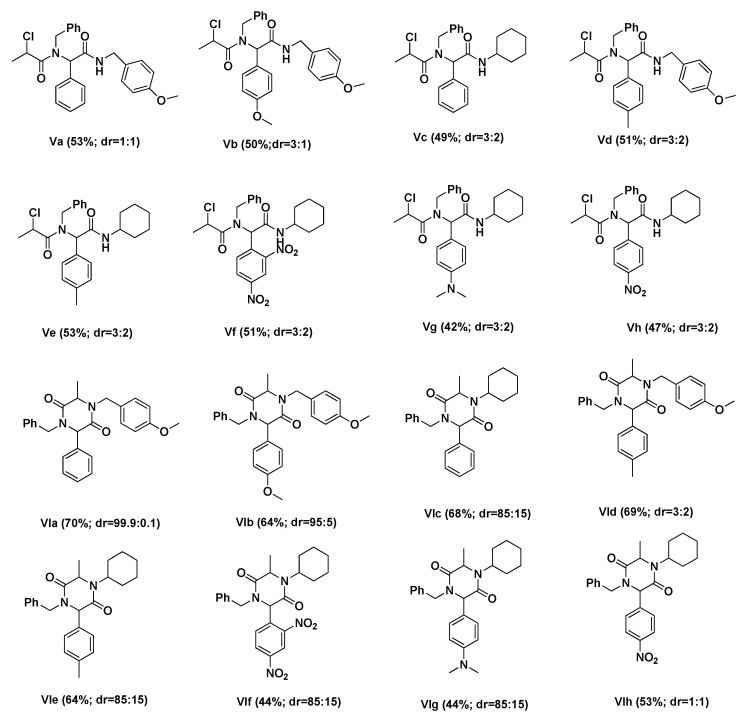
Structures of investigated peptidomimetics and DKPs with isolated yield and diastereomeric ratio in brackets.

Diketopiperazines possess a variety of biological activities. They are found in numerous natural products and are also obtained from the degradation of polypeptides in foodstuff. Their small, conformationally strained structure makes them more attractive along with the possibility of instigating several substituents at six different positions. Due to their rigid backbone, they are quite popular in drug discovery as an important pharmacophore. Post-Ugi transformation for the synthesis of diketopiperazines has been reported [31,32] but these transformations were using reagents such as PPh_3_ which make the purification process hard. In this study, we synthesized a library of differently substituted diketopiperazines varying the substrates for the Ugi multicomponent reaction. To see the effect of substituents on cyclic and acyclic peptidomimetics we used different isocyanides (p-methoxybenzylisocyanide and isocyanocyclohexane) and eight different aldehydes. Diketopiperazines were synthesized in a two-step process. First, target peptidomimetics were obtained via Ugi multicomponent reaction (Scheme 1). A model Ugi reaction was carried out using 2-chloropropionic acid, benzyl amine, benzaldehyde and *p*-methoxybenzylisocyanide as substrates (Scheme 2) in methanol at room temperature following the same procedure reported by us [33], which resulted in product **Va** with 30% yield. To check the effect of temperature on reaction yield we performed the reaction with methanol at 30 °C and obtained product **Va** with 32% yield. A further increase in temperature to 50 °C led to a gradual increase in yield but when the temperature was increased to 60 °C, the reaction yield remained the same which indicates 50 °C as an optimal temperature for this reaction. Since we know that solvent can also affect the progress of the reaction and hence the yield, so we screened various polar protic solvents known for the Ugi reaction and we found Trifluoroethanol to be an efficient solvent for this transformation (Table 1, entry 8). Then we further increased the temperature to 60 °C (entry 9) and yield dropped down to 42%. The mechanistic reason is unclear but it can be that the non-nucleophilic nature of TFE is suppressing the side reactions [34] and increasing the overall yield of product. So, we took the best conditions found in optimization (Table 1, entry 8) for the synthesis of compounds **Va–Vh** with good yield (Figure 2). This Ugi reaction led to the formation of a diastereomeric mixture of peptidomimetics.

**Scheme 3:**
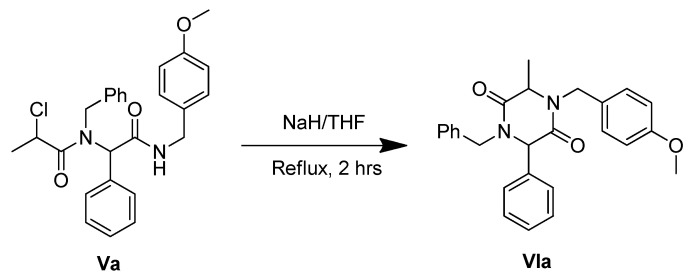
Model cyclisation reaction.

Naliapara et. al. reported the cyclisation of Ugi peptidomimetic using a transition metal catalyst [35]. We modified the conditions for our reaction to eliminate the usage of a metal catalyst (Table 2, Scheme 3, Entry 1). This reaction yielded 25% product. Then, the same reaction was performed at a lower temperature (90 °C; Table 2, entry 2) as well as a high temperature (110 °C; Table 2, entry 3) but yield remained the same. Since the solvent is known to be a crucial parameter in terms of modulating the yield, several different organic solvents were applied (Table 2, entry 4). Further model reaction was conducted at different temperatures (Table 2, entries 4–6); however, it was observed that at high temperatures, the yield decreases (Table 2, entry 6), which may be due to product decomposition. It indicates 65 °C as the optimal temperature for the studied cyclisation reaction. The application of various solvents (Table 2, entry 9–11) resulted in maximum yield (49%) with THF. Having optimized the solvent and temperature for the model reaction, we screened various organic and inorganic bases (Table 2, entry 12–16) and observed that NaH in THF at 65 °C gives product **VIa** with a good yield of 70%. These conditions were further used to obtain DKPs **VIa–VIh** with the yields ranging from 44% to 70%. Upon cyclisation, we observed the change in diastereomeric ratios varying with the attached substituents in the peptidomimetic scaffold.

### 3.2. Cytotoxic Studies of the Synthesized Compounds

**Figure 3:**
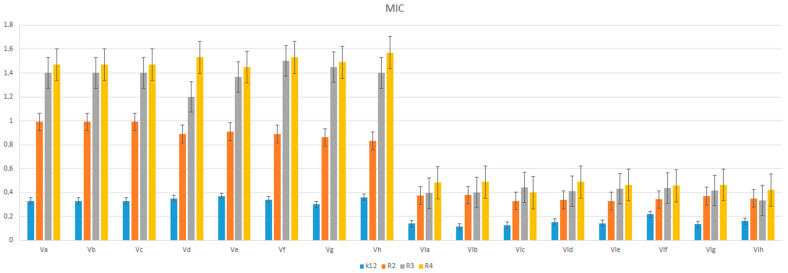
Minimum inhibitory concentration (MIC) of the phosphonate derivatives in model bacterial strains. The *x*-axis features compounds **1**–**16** used sequentially. The *y*-axis shows the MIC value in μg/mL^−1^ Investigated strains of *E. coli* K12 as control (blue), R2 strains (orange), R3 strain (grey), and R4 strain (yellow). The *y*-axis shows the MBC value in μg/mL^−1^. The order in which the compounds were applied to the plate is shown in Supplementary Materials Figure S1.

**Figure 4:**
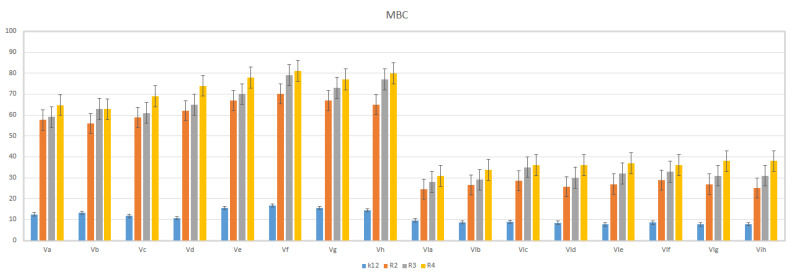
Minimum bactericidal concentration (MBC) of the phosphonate derivatives. The *x*-axis features compounds **Va–Vh and VIa–VIh** used sequentially. The *y*-axis shows the MIC value in μg/mL^−1^. Investigated strains of *E. coli* K12 as control (blue), R2 strains (orange), R3 strain (grey), and R4 strain (yellow). The *y*-axis shows the MBC value in μg/mL^−1^. The order in which the compounds were applied to the plate is shown in Supplementary Materials Figure S1.

**Figure 5:**
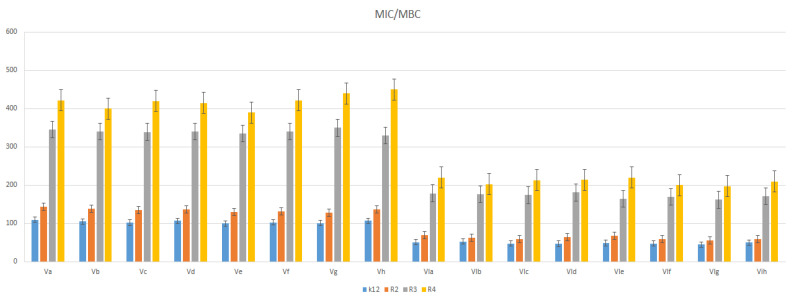
The ratio of MBC/MIC of the phosphonate derivatives. The *x*-axis features compounds **Va–Vh** and **VIa–VIh** used sequentially. The *y*-axis shows the MIC value. Investigated strains of *E. coli* K12 as control (blue), R2 strains (orange), R3 strain (grey), and R4 strain (yellow). The *y*-axis shows the MBC value in μg/mL^−1^. The order in which the compounds were applied to the plate is shown in Supplementary Materials Figure S1.

**Figure 6:**
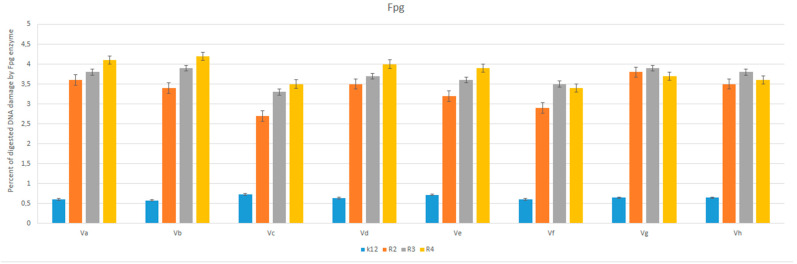
Percentage of plasmid DNA recognized by Fpg enzyme (*y*-axis) with model bacterial, K12, and R2–R4 strains (*x*-axis). All analyzed compounds numbered were statistically significant at <0.05 * (see Table 2 and Supplementary Materials Figure S2 panel A and B).

**Figure 7:**
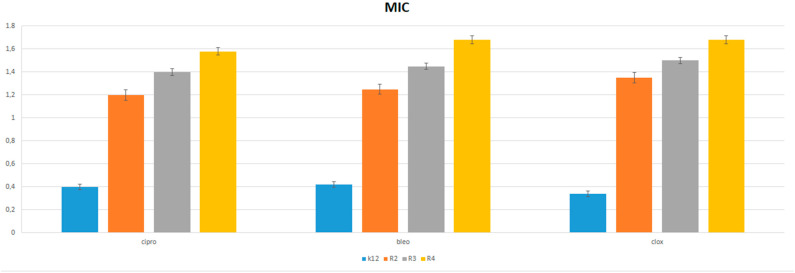
Examples of MIC with model bacterial strains K12, R2, R3, and R4 for studying antibiotics ciprofloxacin (cipro), bleomycin (bleo), and cloxacillin (clox). The *x*-axis features antibiotics used sequentially. The *y*-axis features the MIC value in μg/mL^−1^. Supplementary Materials Figure S1.

The obtained results indicate that all tested Diketopiperazines show cytotoxic activity in all analyzed *E.coli* strains differing in LPS length. Different inhibitory activity was found depending on the nature of the R1 and R2 substituents attached to the chlorine atom of the tested compounds. Among all tested compounds, the compounds from **VIa–VIh** showed a stronger antibacterial effect than **Va–Vh**. It is worth noting that the introduction of the chlorine atom into the structure of the tested compounds had a significant impact on their activity and cytotoxicity and high selectivity against selected *E. coli* model strains in the MIC and MBC tests, which is often observed in various types of compounds showing strong microbiological activity on cells [12]. These compounds showed higher activity against strains R2, R3 and R4 than commonly used antibiotics (Figure 3, Figure 4, Figure 5, Figure 6 and Figure 7). The values of the MIC and MBC tests for each model of *E. coli* R2–R4 and K12 strains were visible on all analyzed growth microplates after the addition of resazurin.

The analyzed bacterial strains used in the experiments were used in 48-well plates; which were treated with the analyzed compounds in the MIC and MBC assays. On the basis of their analysis, color changes were observed for all tested compounds, but at different levels and at different dilutions. The most sensitive to the effects of the analyzed compounds were the bacterial strains R3 and R4 due to the increasing length of their LPS (visible dilutions 10^−2^ corresponding to a concentration of 0.0015 μM); more than strains K12 and R2 (visible dilutions of 10^−6^ corresponding to a concentration of 0.0015 µM). Strain R4 was the most sensitive, possibly due to the longest length of lipopolysaccharide (LPS) in the bacterial membrane. In all analyzed cases, the MBC test values were approximately 75 times higher than the MIC test values in eight analyzed compounds including Cl (Figure 3, Figure 4 and Figure 5 and Table 3).

### 3.3. Analysis of R2–R4 E. coli Strains Modified with Tested Compounds diketopiperazines

**Figure 8:**
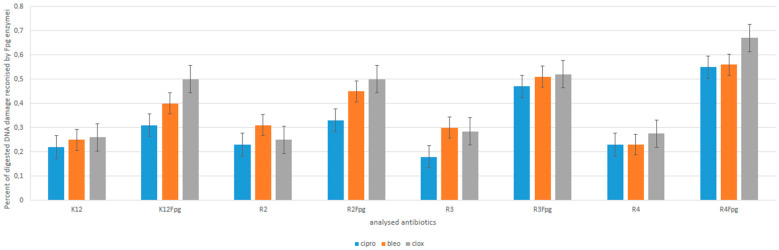
Percentage of bacterial DNA recognized by Fpg enzyme in model bacterial strains after ciprofloxacin, bleomycin, and cloxacillin treatment. The compounds were statistically significant at *p* < 0.05. (See Supplementary Materials Figure S3).

The obtained MIC values, as well as our previous studies with various types of the analyzed compounds [20,21,22,23,24,25,26,27,28,29,30], indicate that derivatives of diketopiperazines also show a strong toxic effect of the analyzed model strains of bacteria. The three compounds analyzed were selected for further analysis by modifying their DNA. Modified bacterial DNA was digested with Fpg as previously described [36,37,38,39,40,41]. All selected analyzed derivatives of diketopiperazines including various types of alkoxy groups, substituents located at aromatic rings and the length of the alkyl chain can strongly change the bacterial DNA topology. After Fpg digestion, approximately 3.5% of the oxidative damage was identified, which, similar to previous observations, indicates very strong oxidative damage in bacterial DNA [7,8,9]. Different types of alkoxy groups, substituents located on the aromatic ring and the length of the alkyl chain, may determine the toxicity of the analyzed *E. coli* strains, including in particular R4, as evidenced by the obtained MIC, MBC and MTT values. The obtained results for individual compounds were statistically significant at the level of *p* < 0.05 (Figure 6, Figure 7 and Figure 8).

### 3.4. R2–R4 E. coli Strains with Tested Peptidomimetics

The performed studies support the concept that the synthesized compounds can be considered drug candidates for further studies (Supplementary Materials Figure S3).

## 4. Conclusions

An efficient method for the synthesis of peptidomimetics was developed using the Ugi reaction; these were then used to synthesis diketopiperazines. The established protocol was used to synthesize a series of target products containing differently substituted aldehydes and isocyanides. This protocol ensures the efficient, gentle and metal-free synthesis of the target products with good yields (49%–70%). The cytotoxic effect of the obtained cyclic and acyclic peptidomimetics was assessed on model *E. coli* strain and its mutants. The analyzed diketopiperazines derivatives are able to modify all model strains of *E. coli* (R2–R4) and their bacterial DNA, changing the spatial structure of LPS contained in their cell membranes. Compared to the derivatives, the most active among the tested derivatives turned out to be those with cyclic structures **VI**–**VIh**. We have found that the stiffening of the peptidomimetic structure is responsible for increase in their antimicrobial activity. Figure 3 reveals that compounds **VIa**, **VIb**, **VIc** and **VIg** have the highest potential as anti-bacterial drug candidates among the tested DKPs. Our studies also show that synthesized DKPs have a lower MIC value compared to well-known antibiotics, which allows us to say that DKPs hold more potential as antibiotic drug candidates due to high anti-bacterial activity for all the tested mutants. The results of the presented research are important for understanding the biological properties of the studied derivative diketopiperazines as a function of potential new antibiotics and their toxic effects on gram-negative bacteria and cancer cell lines in the face of the growing drug resistance pandemic. Referring to our previous work related to the characteristics of the *E. coli* K12 and R2–R4 models it is worth continuing this research on the separation of diastereomers of the analyzed compounds and their studies on *E. coli* strains and on human cell lines in various types of cancer.

## Figures and Tables

**Table 1:** Influence of solvent on model Ugi reaction.

Entry	Solvent	Temperature (°C)	Yield (%)
1	Methanol	25	30
2	Methanol	30	32
3	Methanol	40	33
4	Methanol	50	39
5	Methanol	60	39
6	Ethanol	50	33
7	Isopropanol	50	37
8	TFE	50	53
9	TFE	60	42

**Table 2:** Optimization for model cyclisation reaction.

Entry	Base	Solvent	Temperature	Yield (%)
1	K_2_CO_3_	DMF	100	25
2	K_2_CO_3_	DMF	90	27
3	K_2_CO_3_	DMF	110	26
4	K_2_CO_3_	Ethanol	50	30
5	K_2_CO_3_	Ethanol	65	39
6	K_2_CO_3_	Ethanol	75	28
7	K_2_CO_3_	THF	65	49
8	K_2_CO_3_	DMF	65	30
9	K_2_CO_3_	DMSO	65	32
10	K_2_CO_3_	Toluene	65	29
11	K_2_CO_3_	Methanol	65	34
12	CsF	THF	65	33
13	NaHCO_3_	THF	65	36
14	KO^t^Bu	THF	65	27
15	KOH	THF	65	19
16	NaH	THF	65	70

**Table 3:** Statistical analysis of all analyzed compounds by MIC, MBC, and MBC/MIC; <0.05 *, <0.01 **, <0.001 ***.

No. of Samples	Va	Vb	Vc	Vd	Ve	Vf	Vg	Vh	Type of Test
K12	**	**	**	**	*	*	*	**	MIC
R2	**	**	**	**	*	*	*	**	MIC
R3	**	**	**	**	*	*	*	**	MIC
R4	**	**	**	**	*	*	*	**	MIC
K12	*	*	**	*	**	*	*	**	MBC
R2	*	*	**	*	**	*	*	**	MBC
R3	*	*	**	*	**	*	*	**	MBC
R4	*	*	**	*	**	*	*	**	MBC
K12	**	*	*	*	*	*	*	***	MBC/MIC
R2	**	*	*	*	*	*	**	***	MBC/MIC
R3	**	*	*	*	*	*	**	***	MBC/MIC
R4	**	*	*	*	*	*	**	***	MBC/MIC

## Data Availability

On request of those interested.

## Supplementary Materials

The following supporting information can be downloaded at: https://www.mdpi.com/article/10.3390/molecules27113633/s1, Figure S1. Examples of MIC and MBC on microplates with different concentration of studied compounds (mg L^−1^). Figure S2. An example of an agarose gel electrophoresis separation of isolated plasmids DNA on R4 strains modified with selected compounds (Panel A) from 8 selected compounds, as shown in Figure 3, and digested with repair Fpg protein (Panel B). M = marker. Figure S3. Example of an agarose gel electrophoresis separation of isolated plasmids DNA from R2–R4 strains modified with antibiotics: bleomycin, ciprofloxacin, and cloxacillin digested with repair enzymes Fpg. M = marker. Figure S4: Structure of antibiotics. Figure S5: NMR spectra of synthesized compounds **Va–Vh & VIa–VIh**.

## Sample Availability

On request of those interested.

## Abbreviations

MIC	minimum inhibitory concentration
MBC	minimum bactericidal concentration
Oc	open circle
Ccc	covalently closed circle
BER	base excision repair
Fpg	DNA-formamidopyrimidine glycosylase
