# Supplementary material for: Influence of Open Chain and Cyclic Structure of Peptidomimetics on Antibacterial Activity in E. coli Strains
Source: Molecules. 2022 Jun 6;27(11):3633. doi: 10.3390/molecules27113633 (PMC9182016; doi:10.3390/molecules27113633)
Supplement: Supplementary file 1 [file molecules-27-03633-s001.zip › molecules-1734804-supplementary.pdf]

Supplementary materials

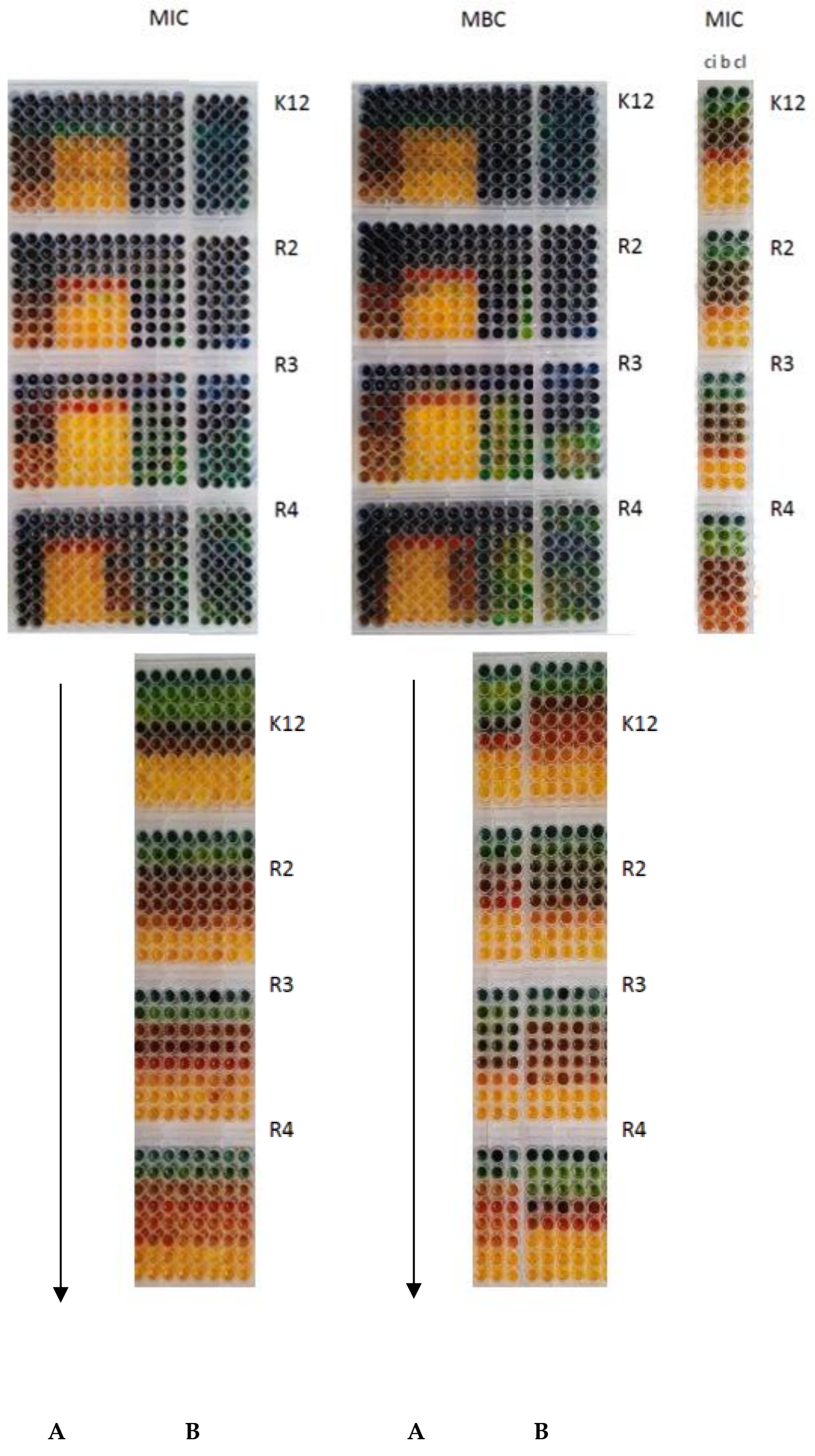

Figure S1. Examples of MIC and MBC on microplates with different concentration of studied compounds (μg/mL⁻¹). Resazurin was added as an indicator of microbial growth with K12, R2, R3, and R4 strains with tested 16 compounds. **A panel indicated by black arrows MIC dilution 8 times for first 8 compounds with Cl, B panel MIC dilution 16 times for another one 8 compounds without Cl** Additionally, examples of MIC with different strains K12, R2, R3, and R4 of studied antibiotics with ciprofloxacin (ci), bleomycin (b), and cloxacillin (cl) in (μg/mL⁻¹).

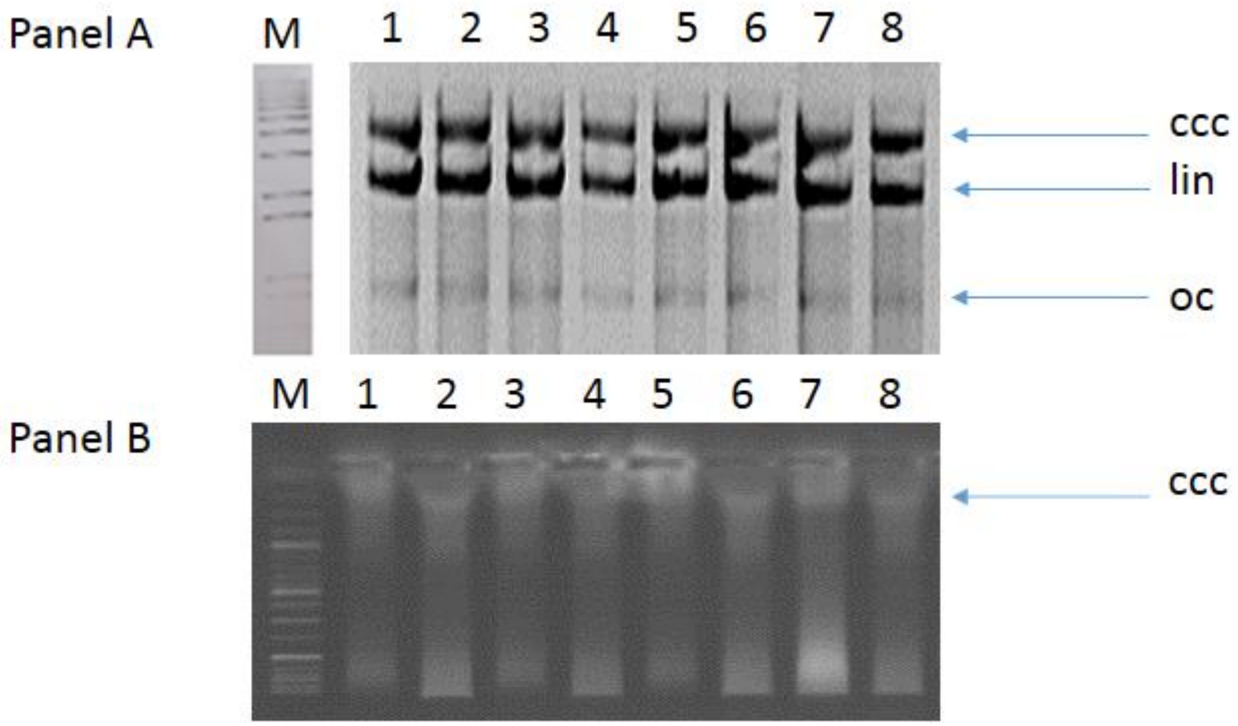

FigureS2. An example of an agarose gel electrophoresis separation of isolated plasmids DNA on R4 strains modified with selected coumarin derivatives (Panel A) from 8 selected compounds, as shown in Figure 3, and digested with repair Fpg protein (Panel B). M = marker.

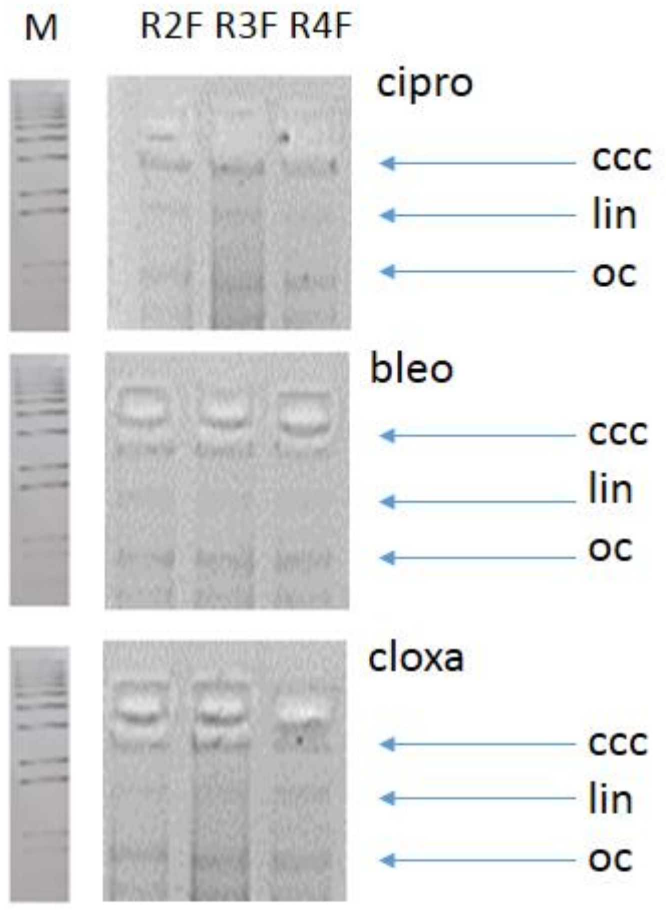

Figure S3. Example of an agarose gel electrophoresis separation of isolated plasmids DNA from R2-R4 strains modified with antibiotics: bleomycin, ciprofloxacin, and cloxacillin digested with repair enzymes Fpg. M = marker.

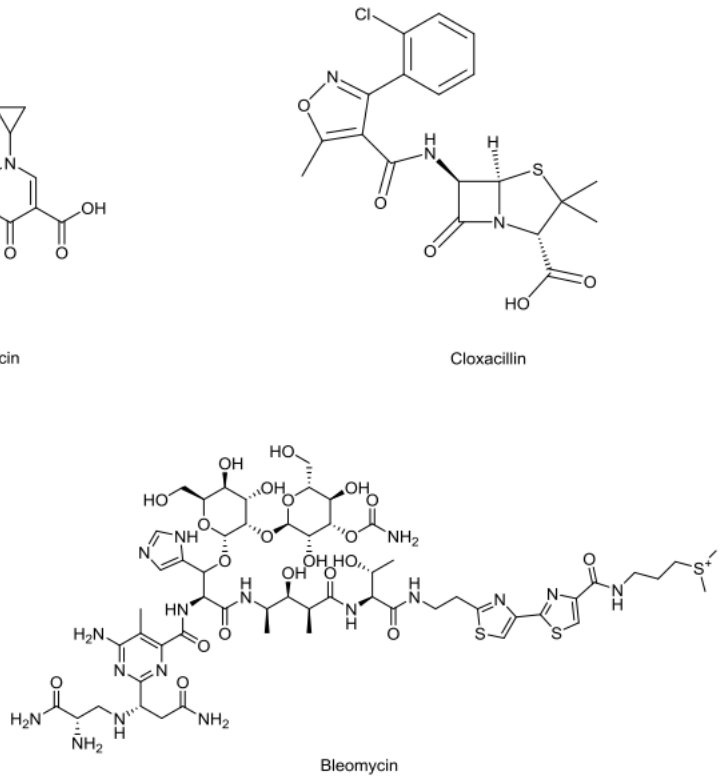

Figure S4: Structure of studied antibiotics

Compound **Va**: ^{1}H NMR

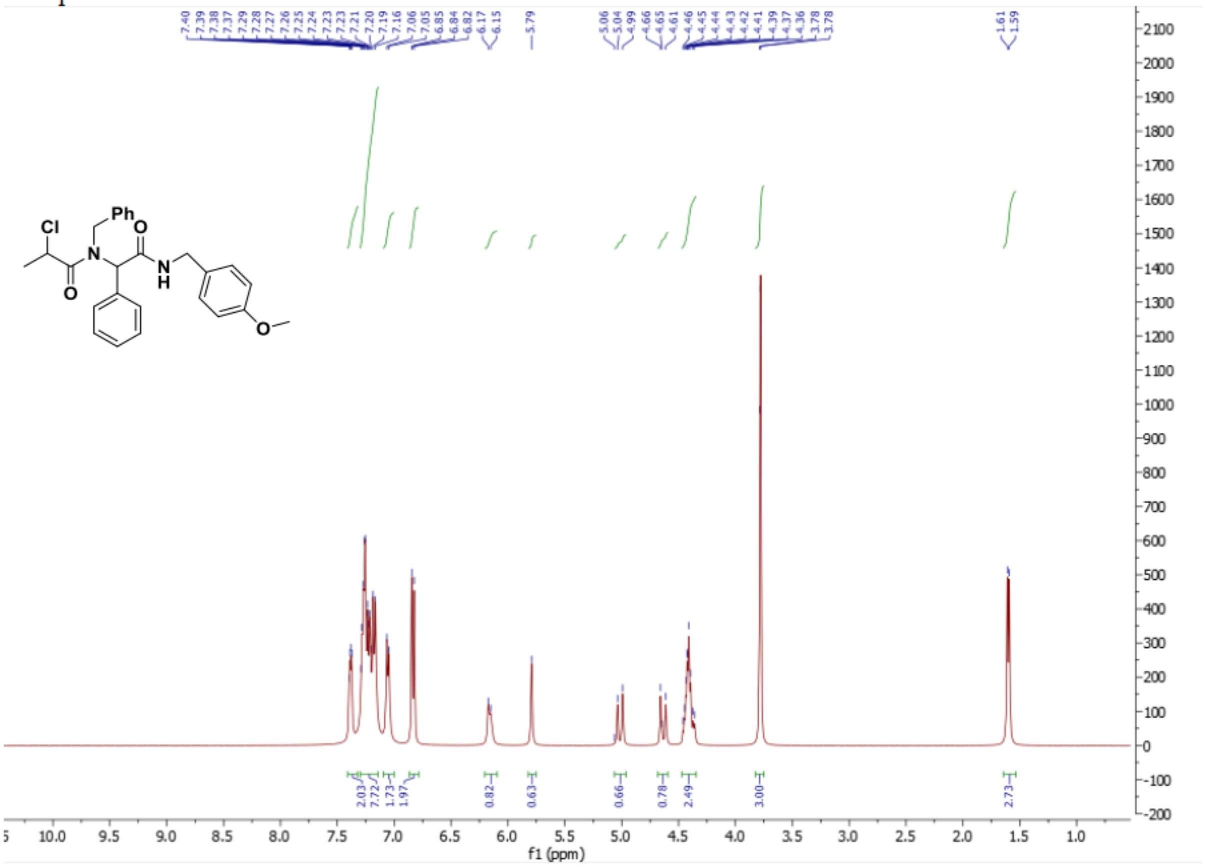

^{13}C NMR:

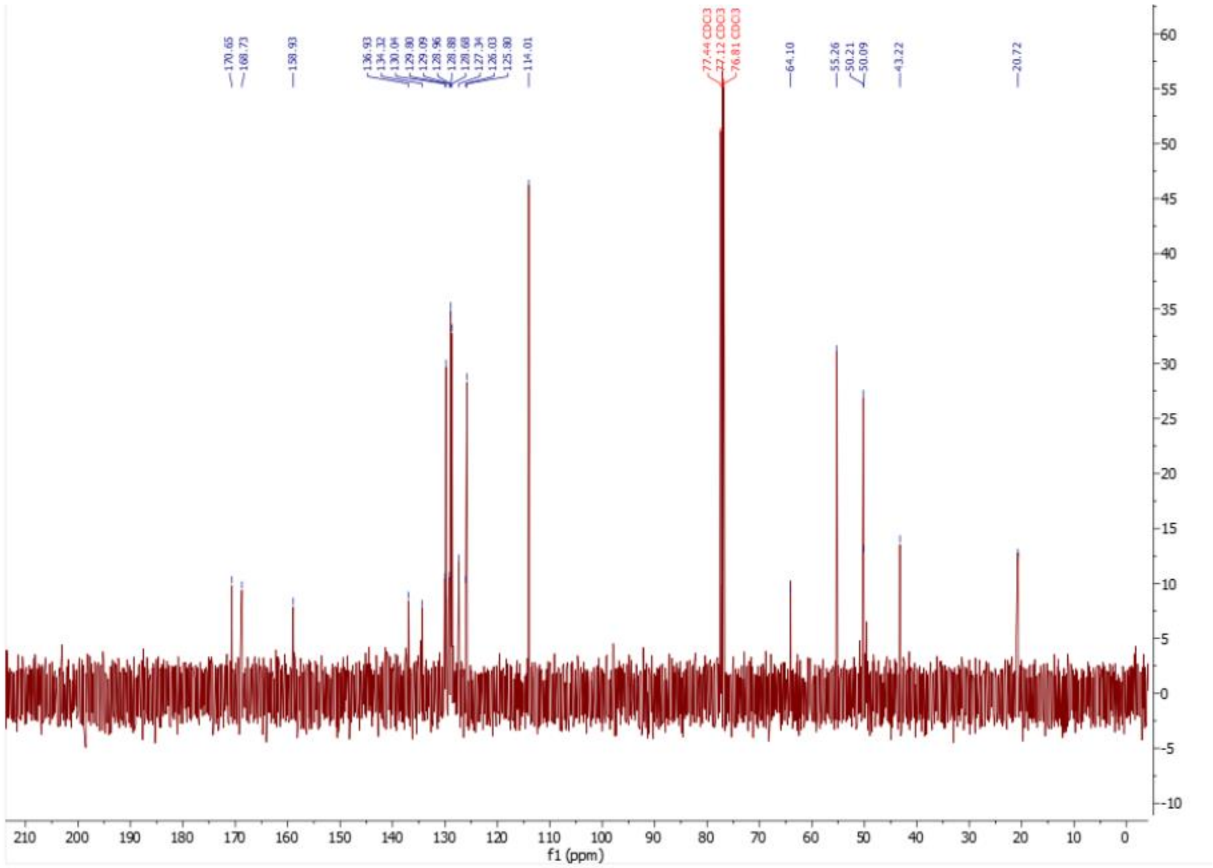

Compound **Vb**: ^{1}H NMR

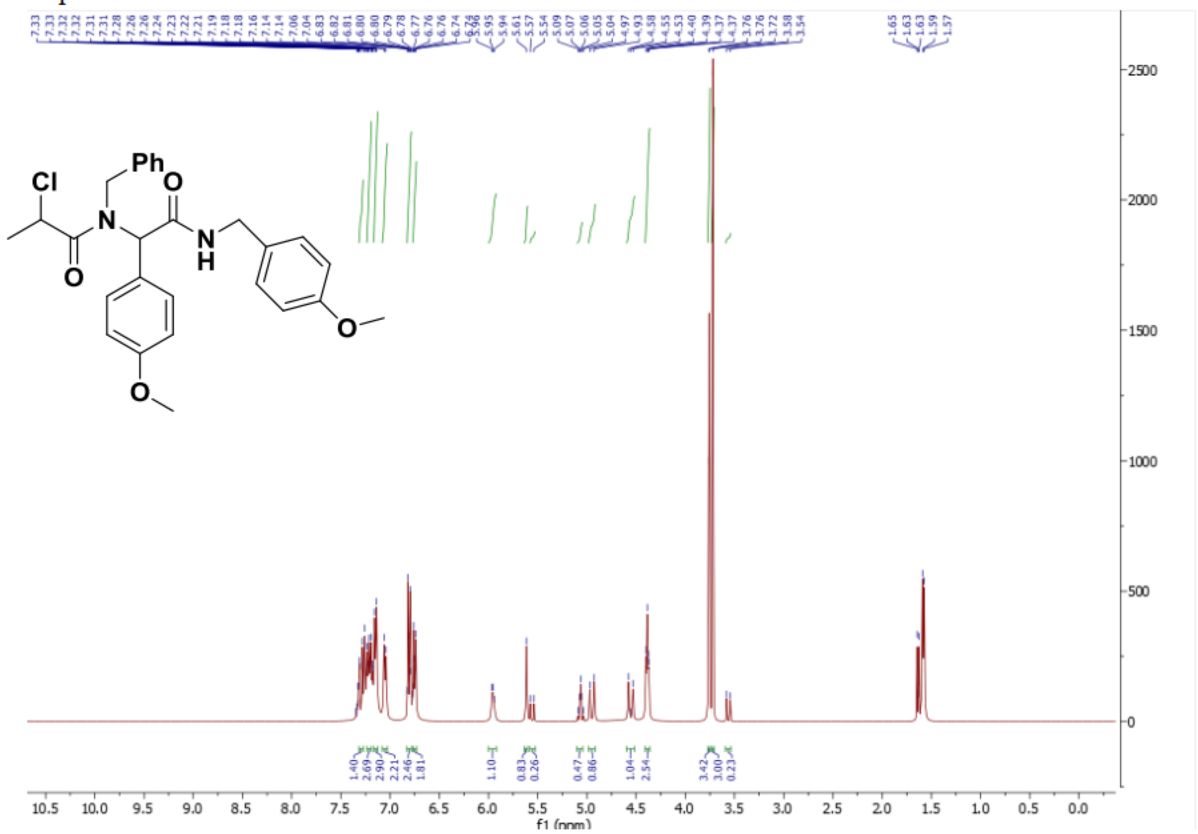

^{13}C NMR:

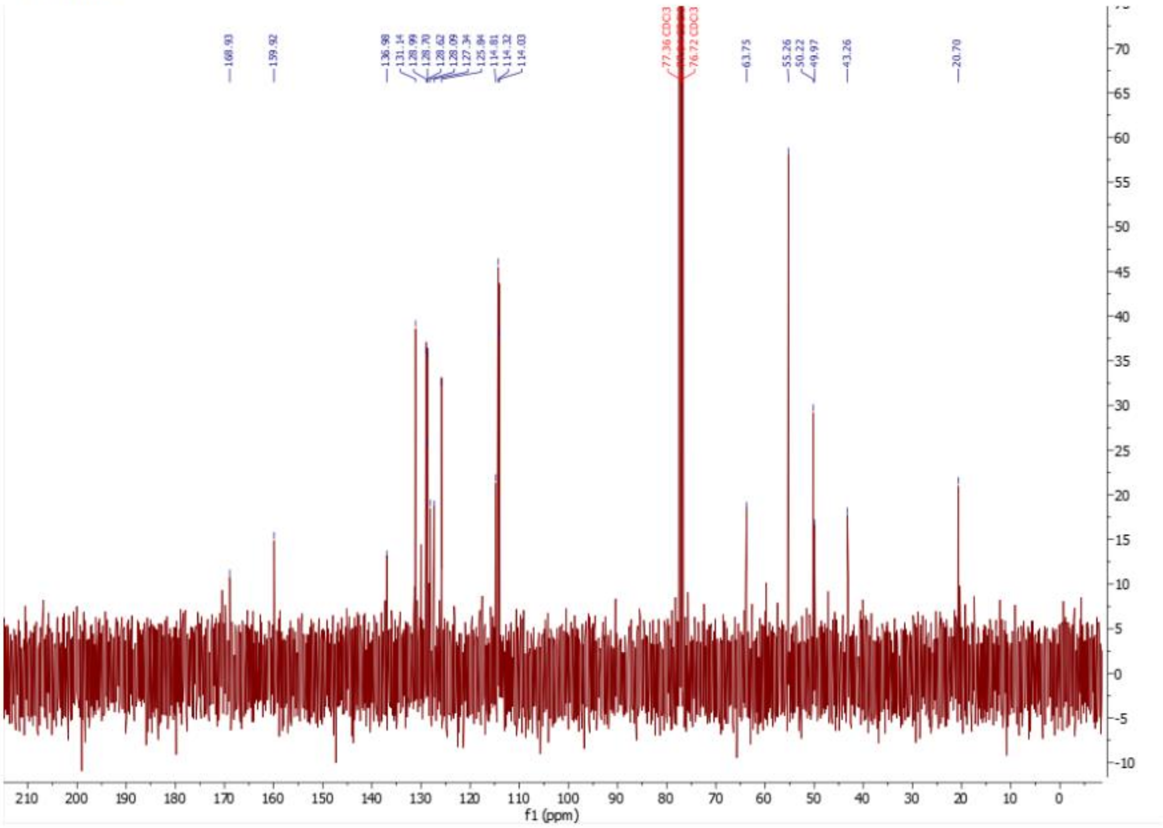

Compound **Vc:**

^{1}H NMR:

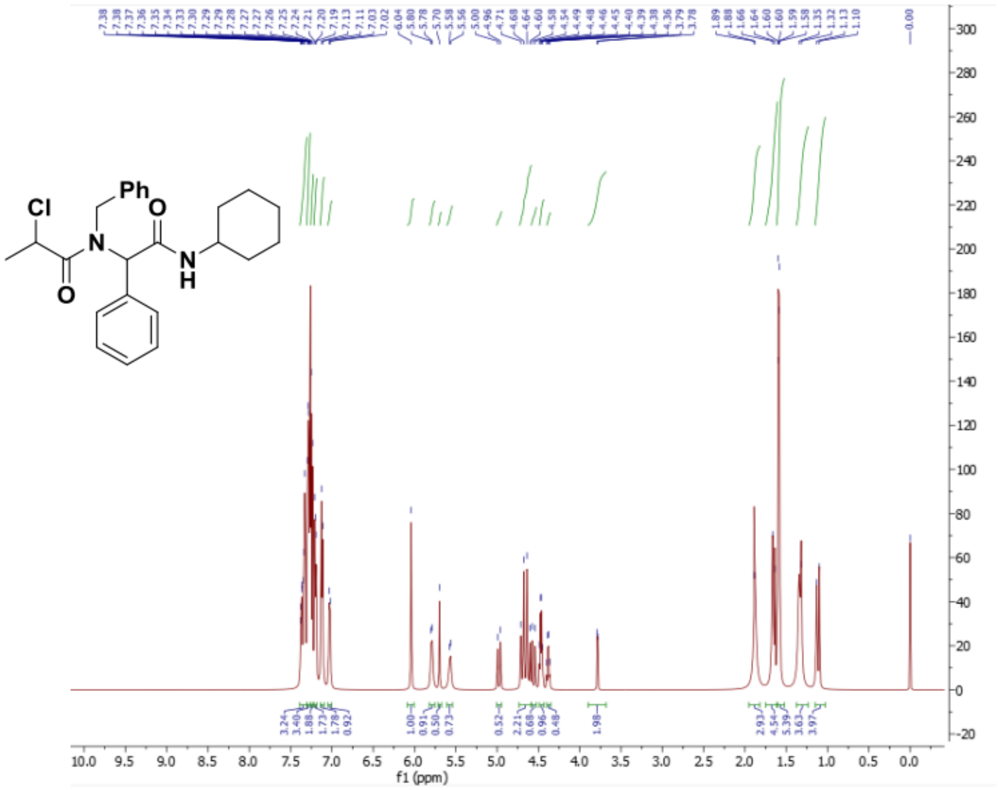

^{13}C NMR:

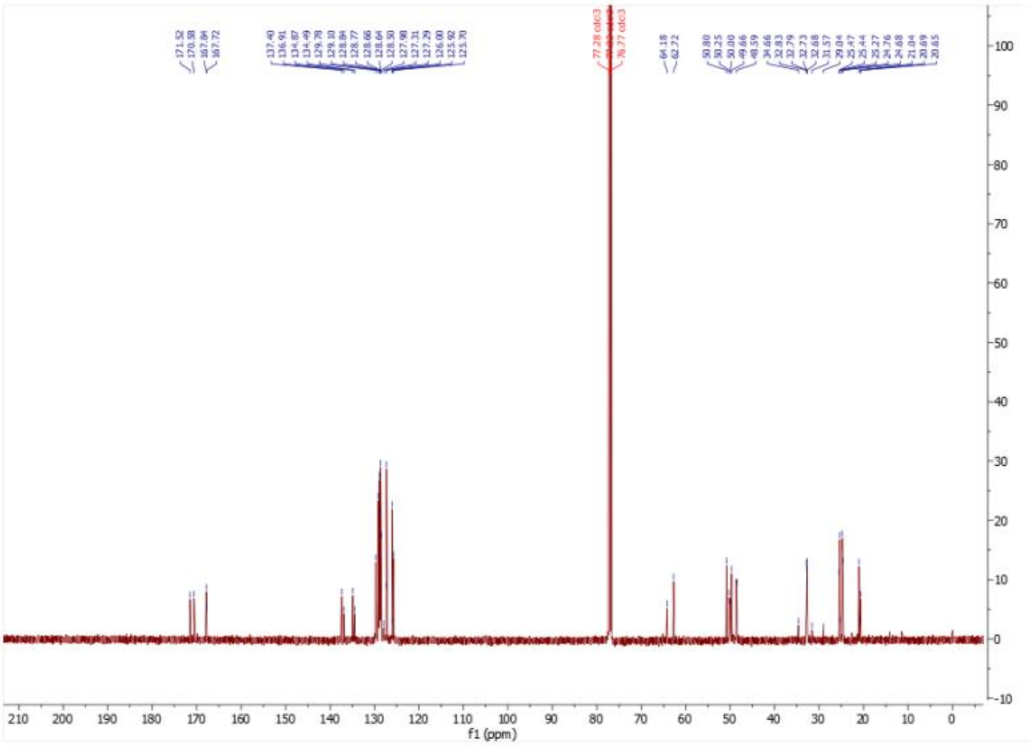

Compound **Vd:**

^{1}H NMR:

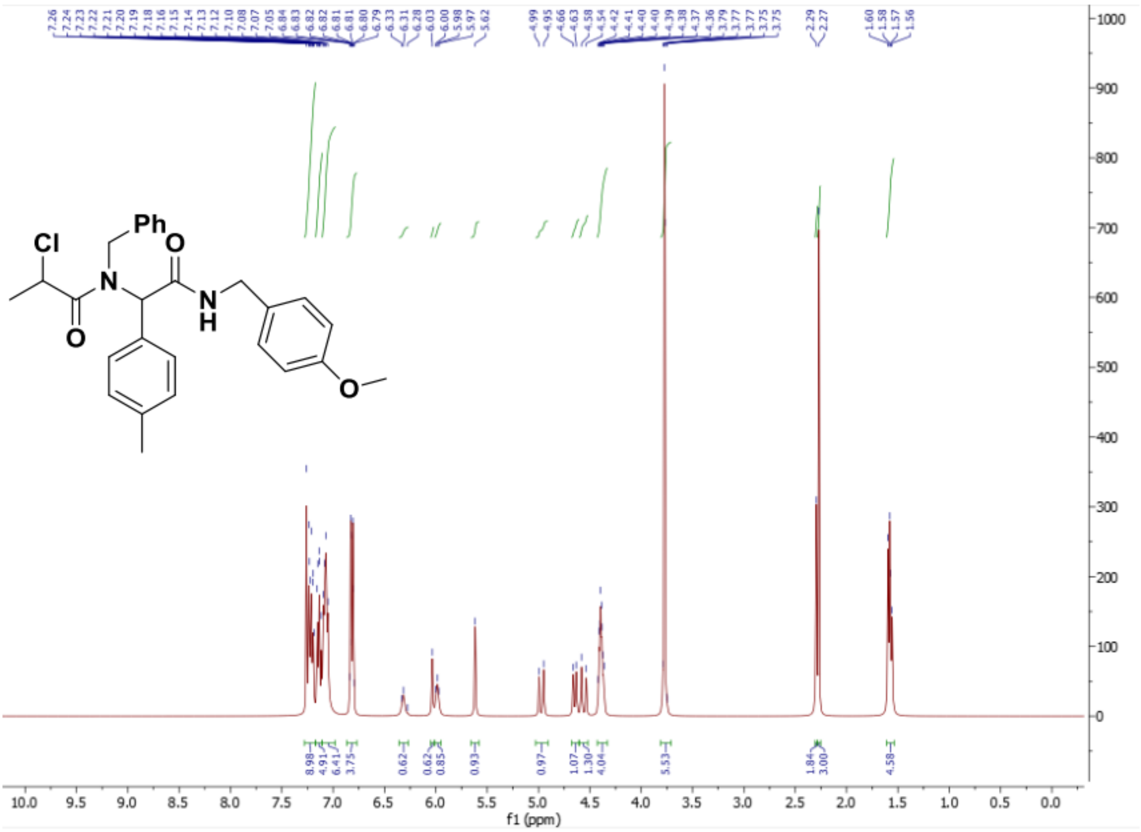

^{13}C NMR:

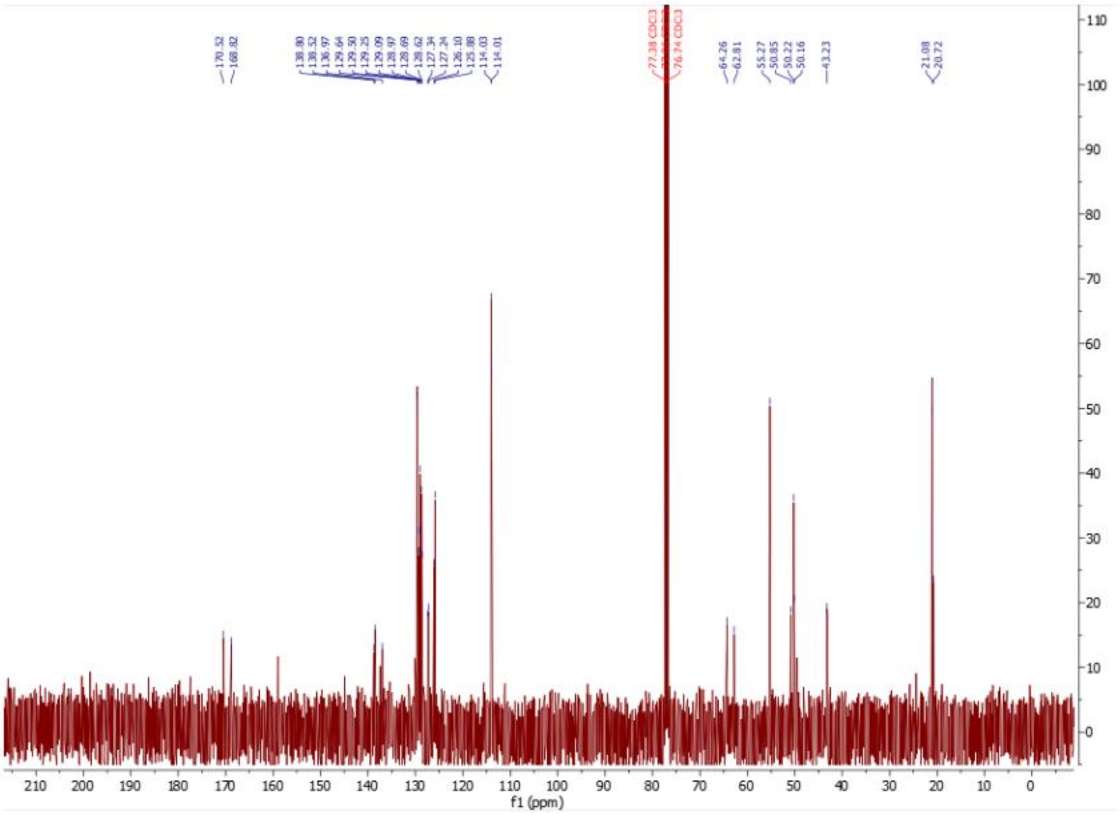

Compound **Ve:**

^{1}H NMR:

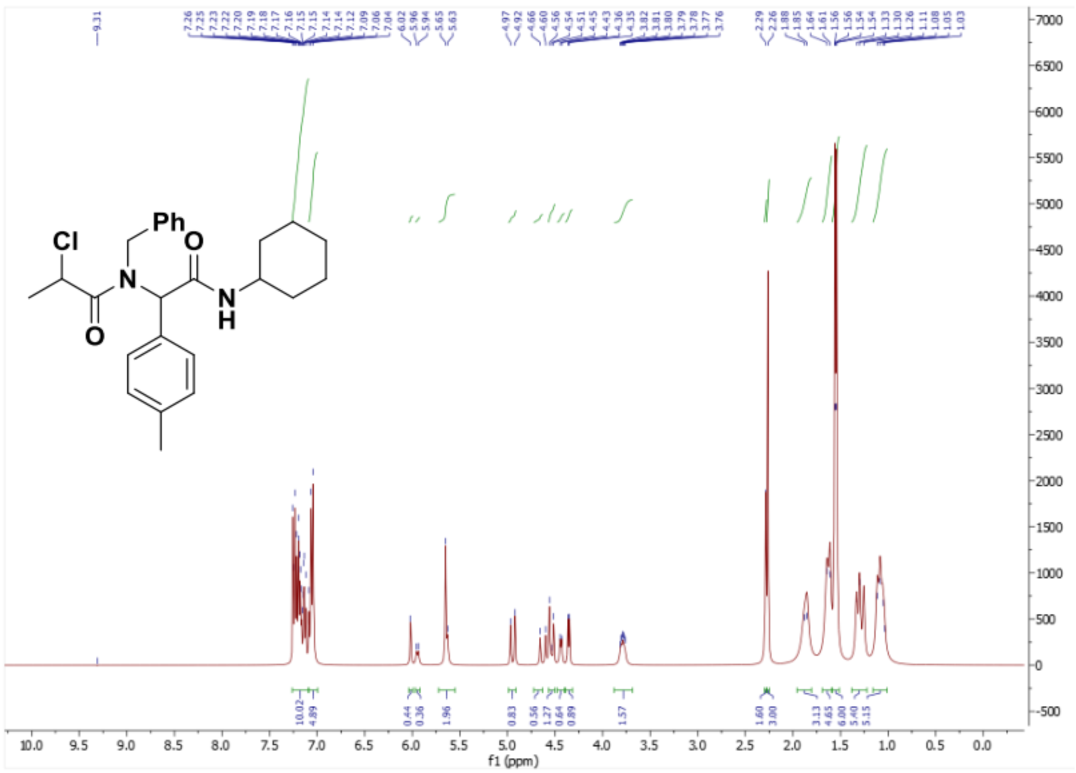

^{13}C NMR:

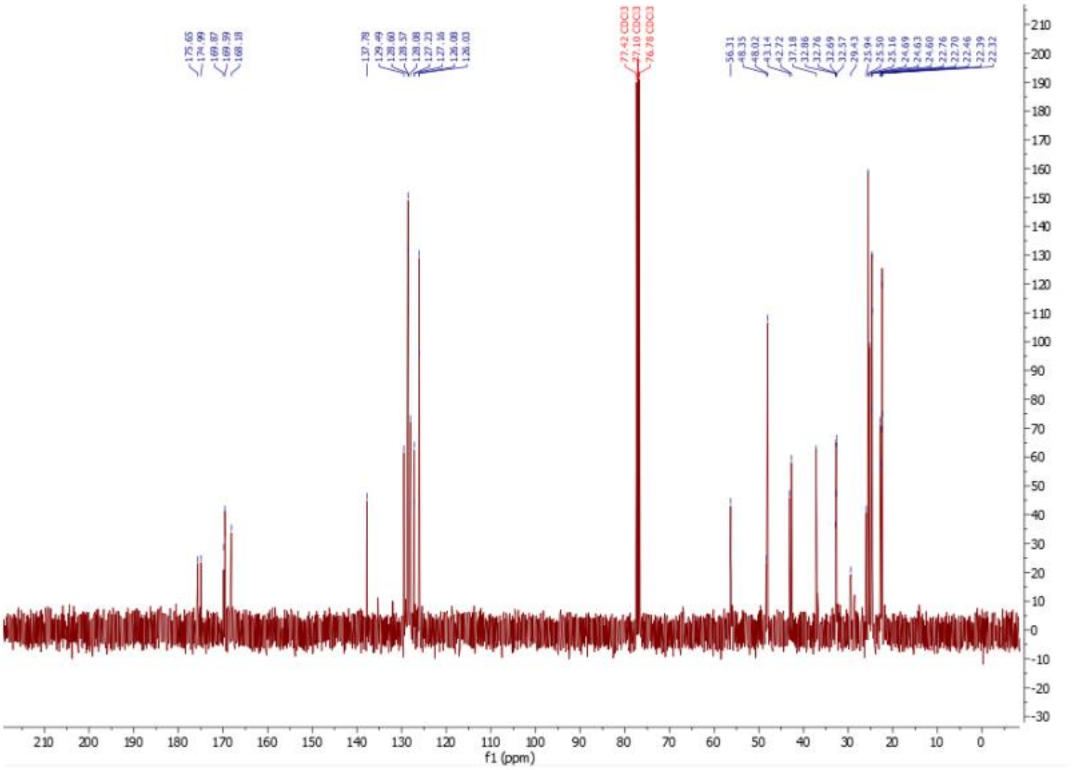

Compound **Vf:**

^{1}H NMR:

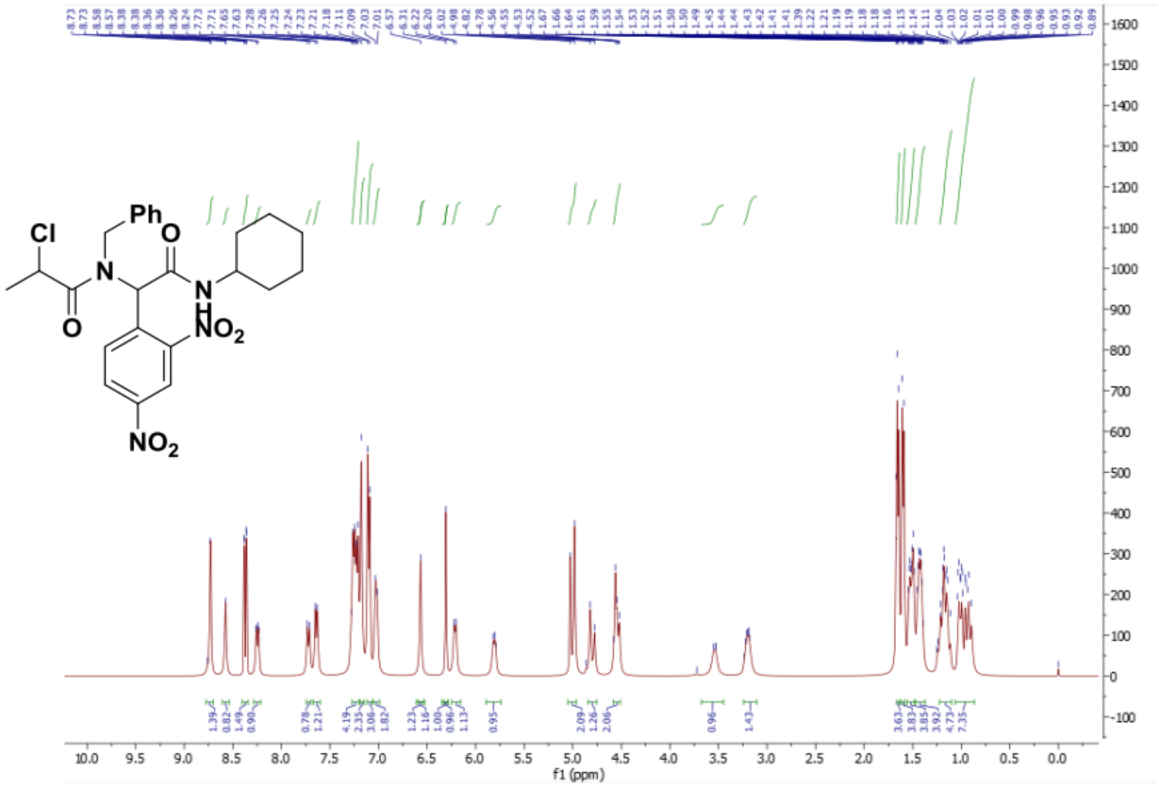

^{13}C NMR:

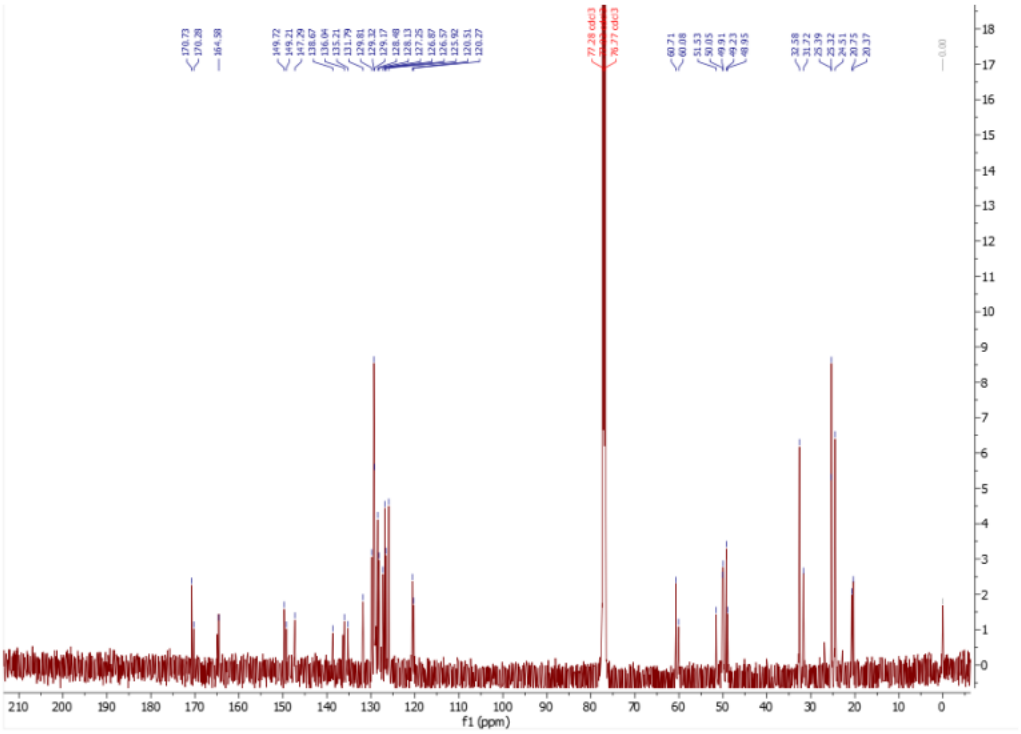

Compound **Vg:**

^1H NMR:

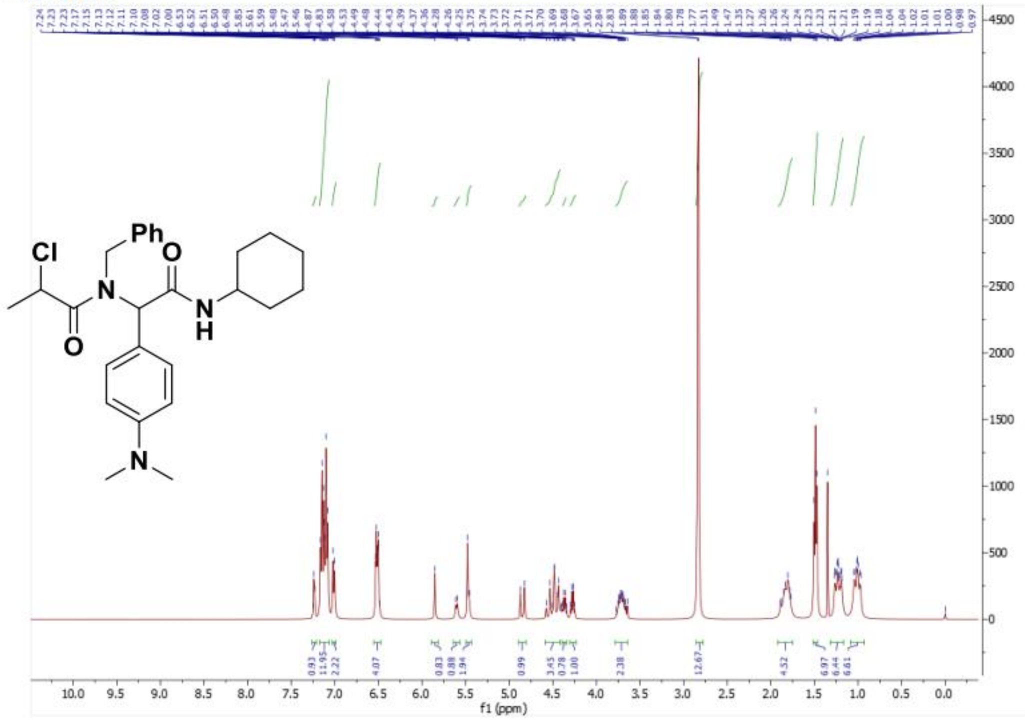

13C NMR:

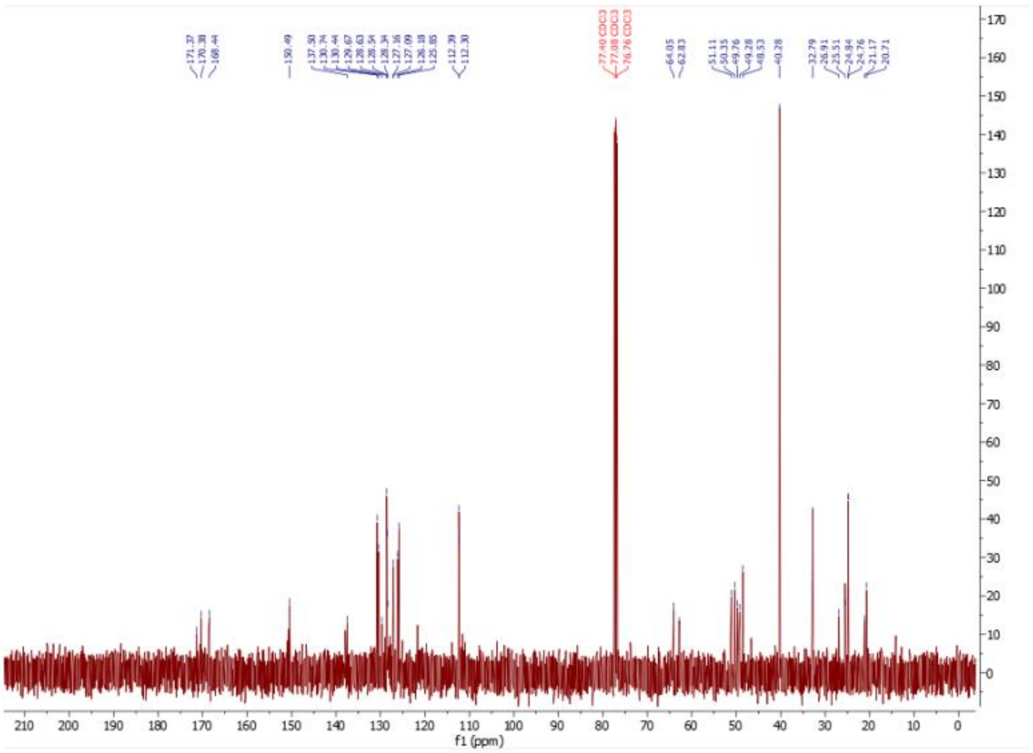

Compound **Vh:**

1H NMR:

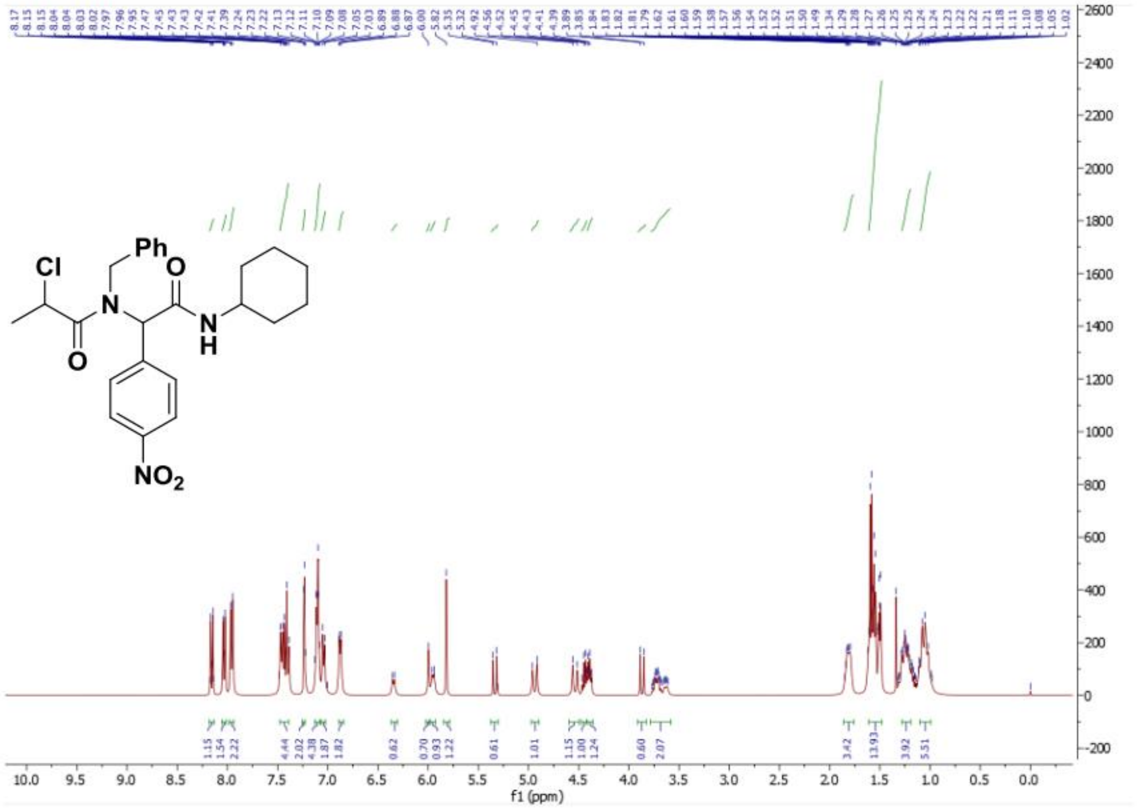

^{13}C NMR:

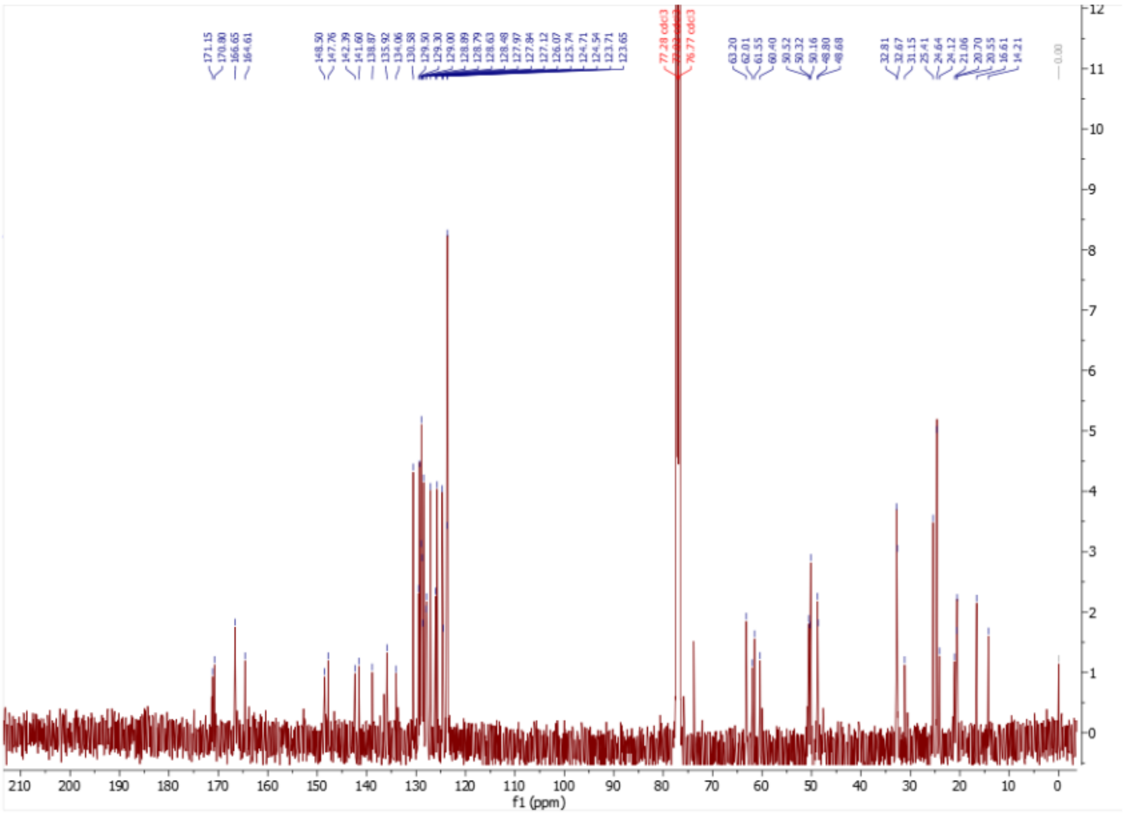

Compound **VIa**:

^{1}H NMR:

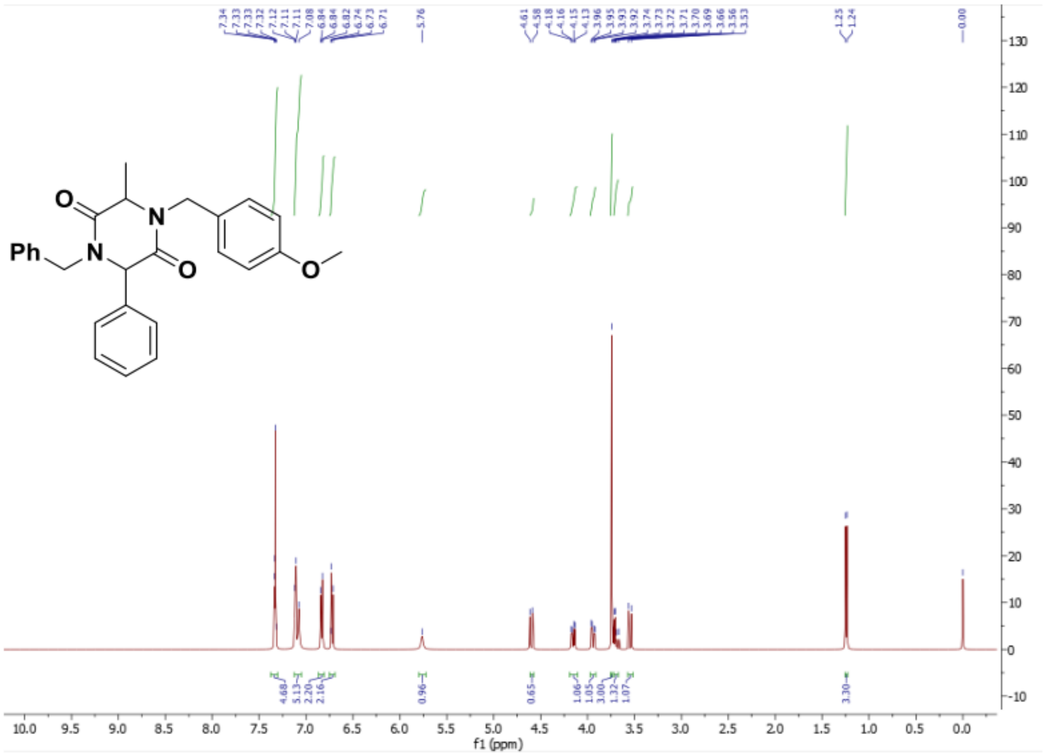

^{13}C NMR:

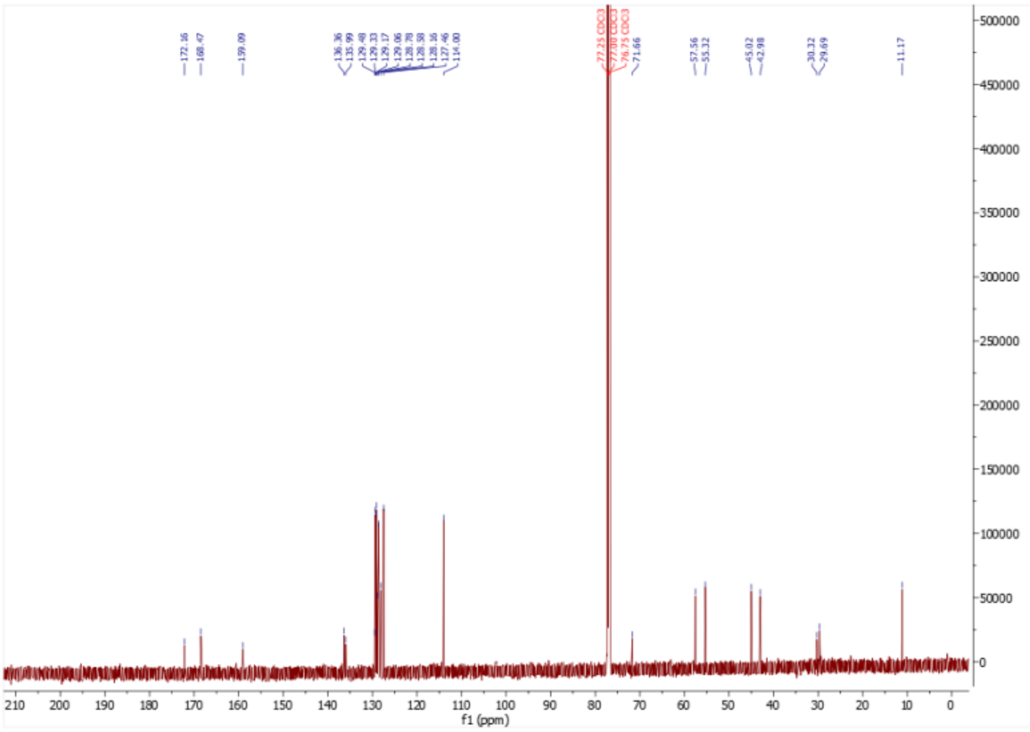

Compound **VIb:**

^{1}H NMR:

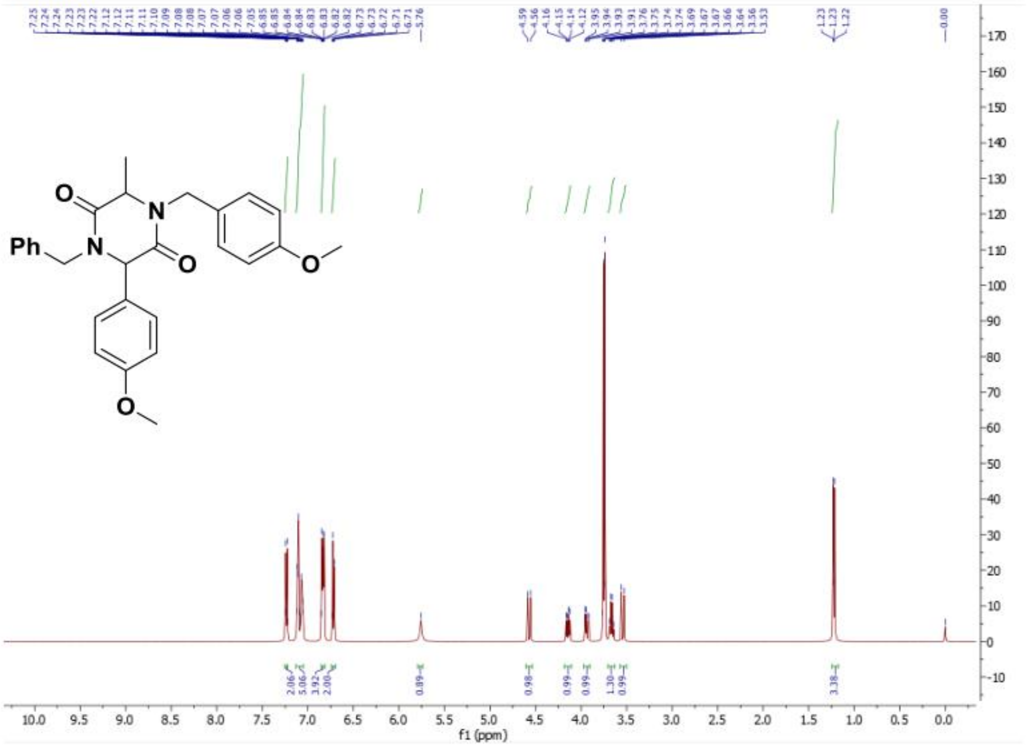

^{13}C NMR:

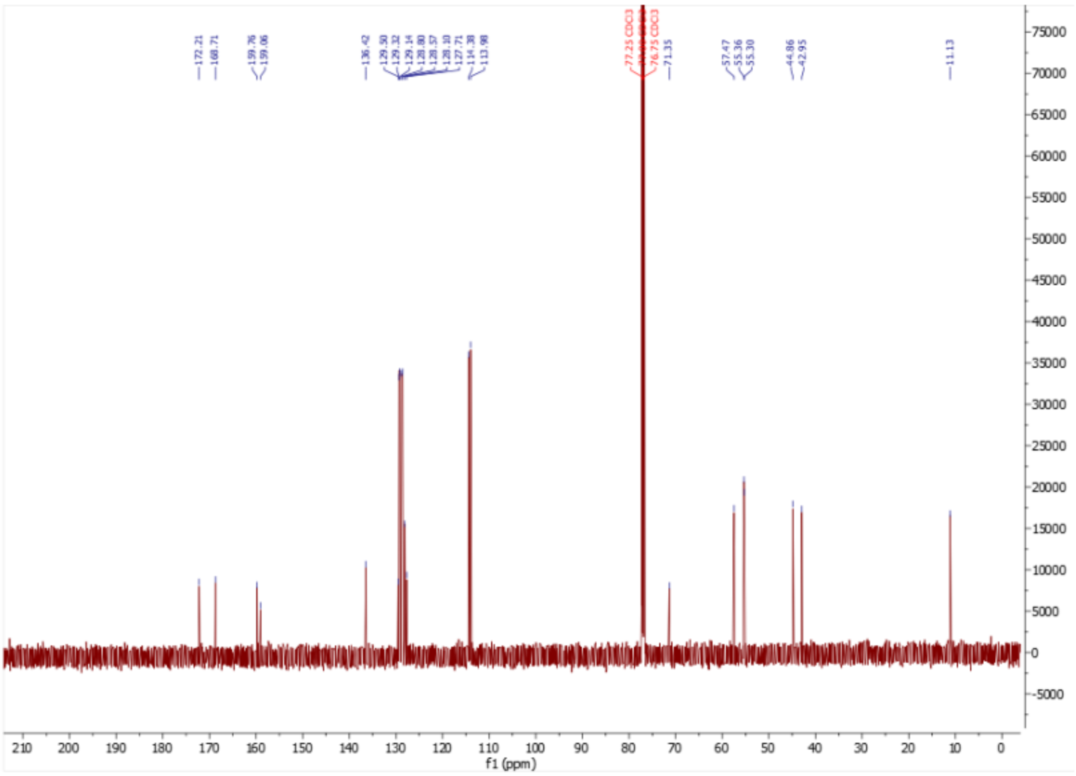

Compound **VIc:**

^{1}H NMR:

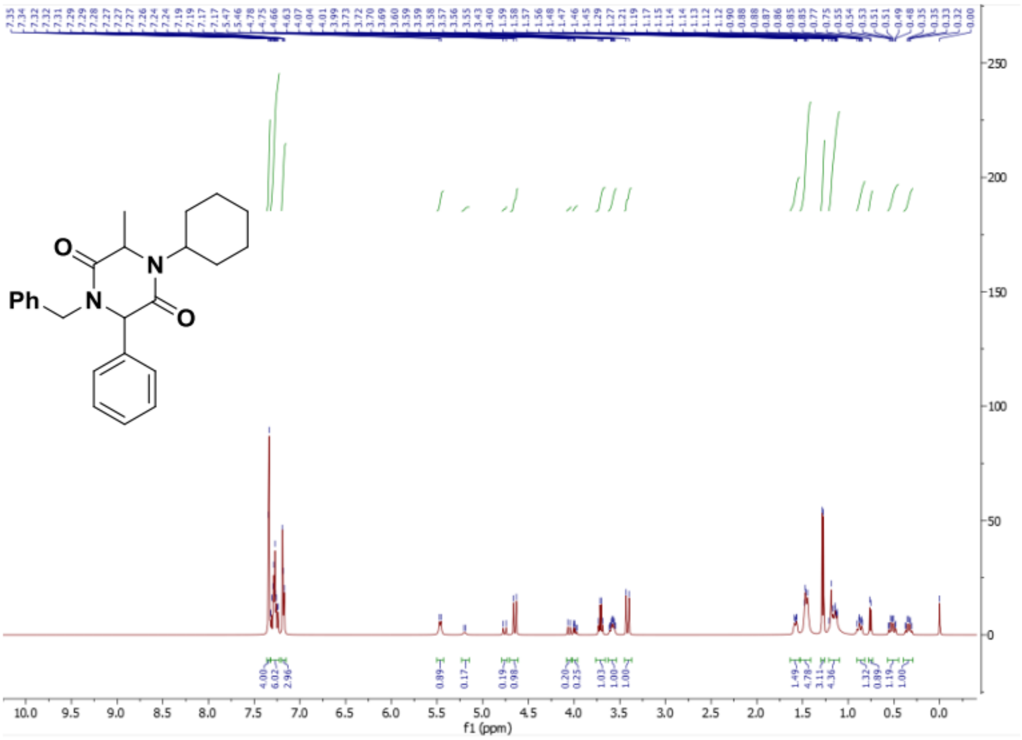

^{13}C NMR:

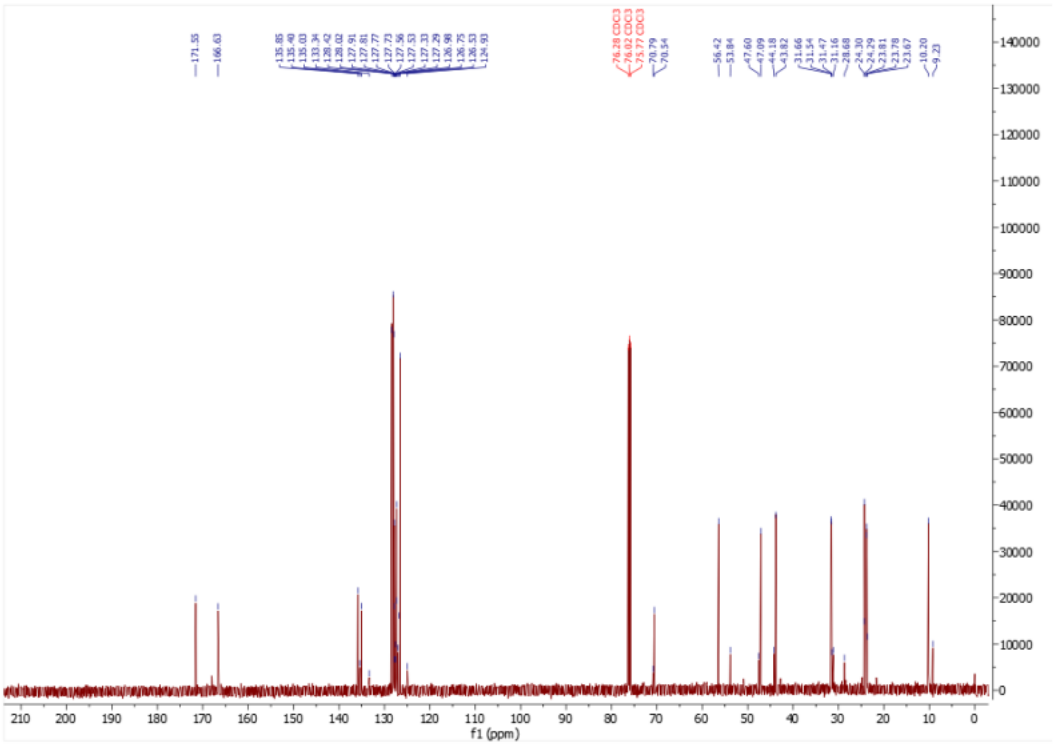

Compound **VId:**

^{1}H NMR:

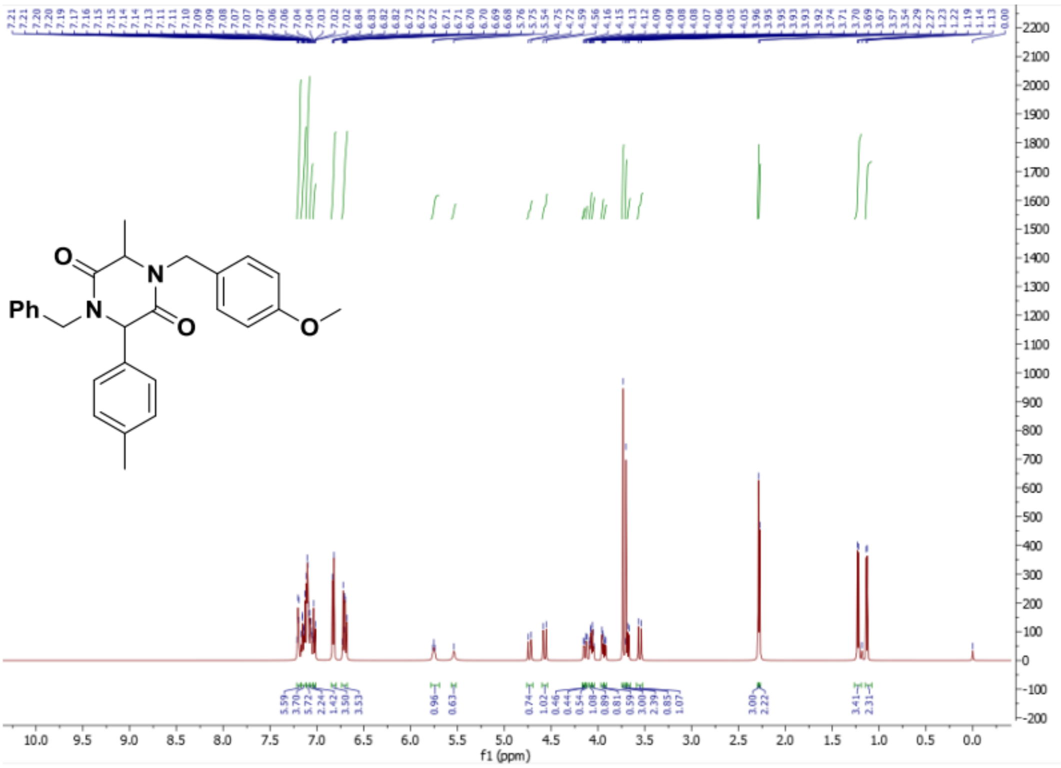

^{13}C NMR:

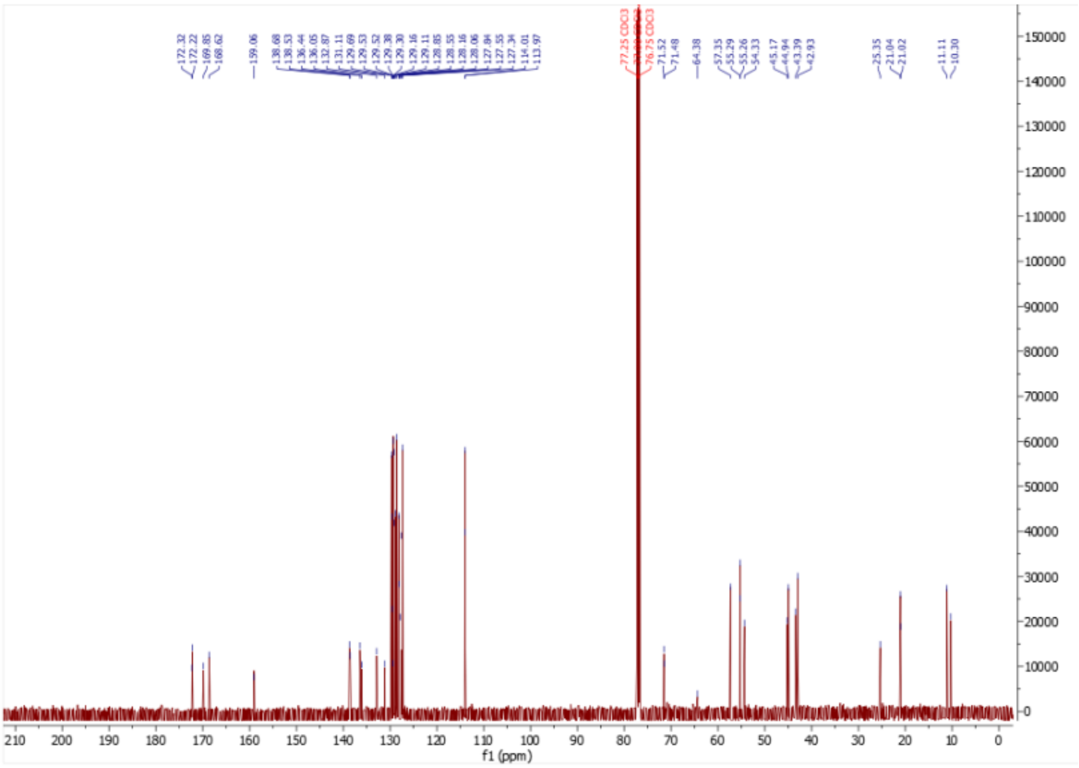

Compound **VIe:**

^{1}H NMR:

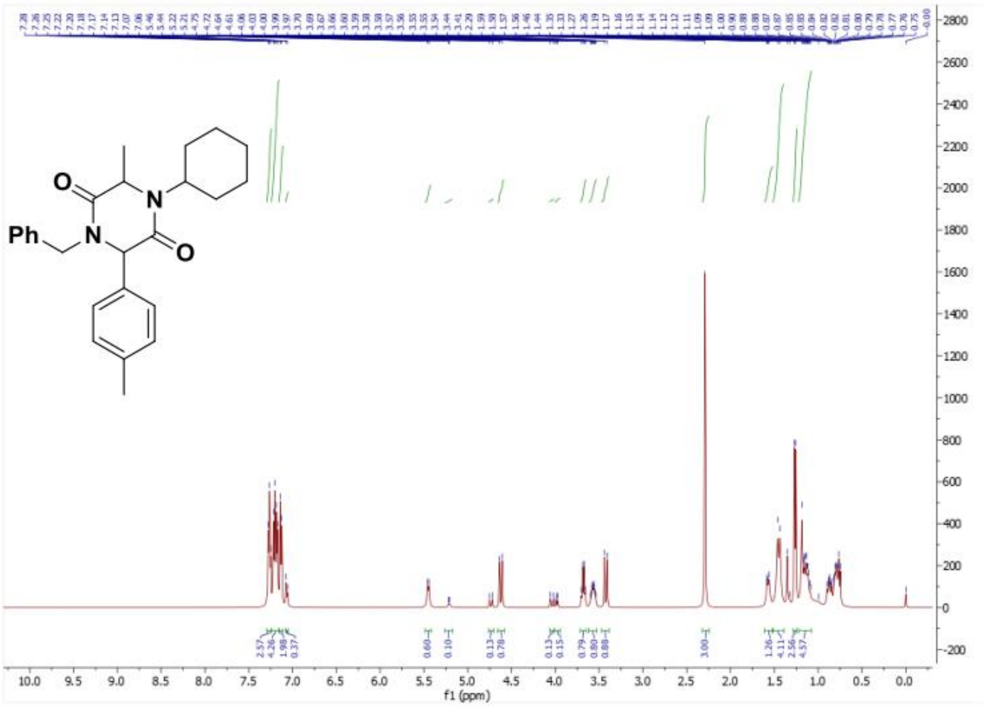

^{13}C NMR:

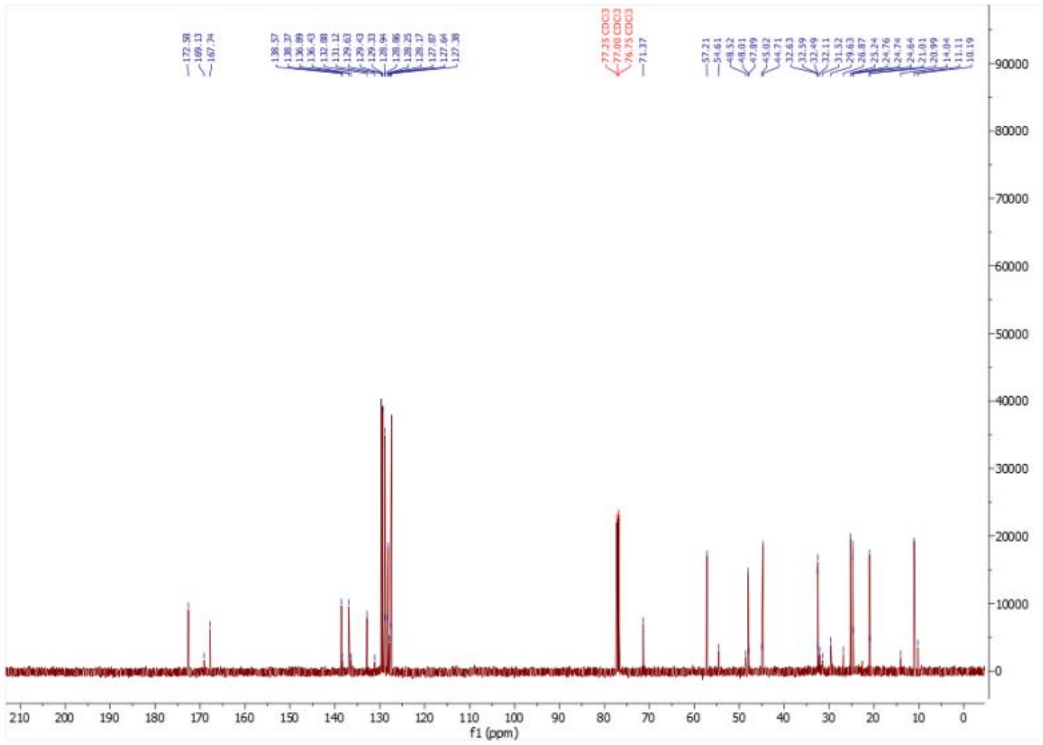

Compound **VIf:**

^{1}H NMR:

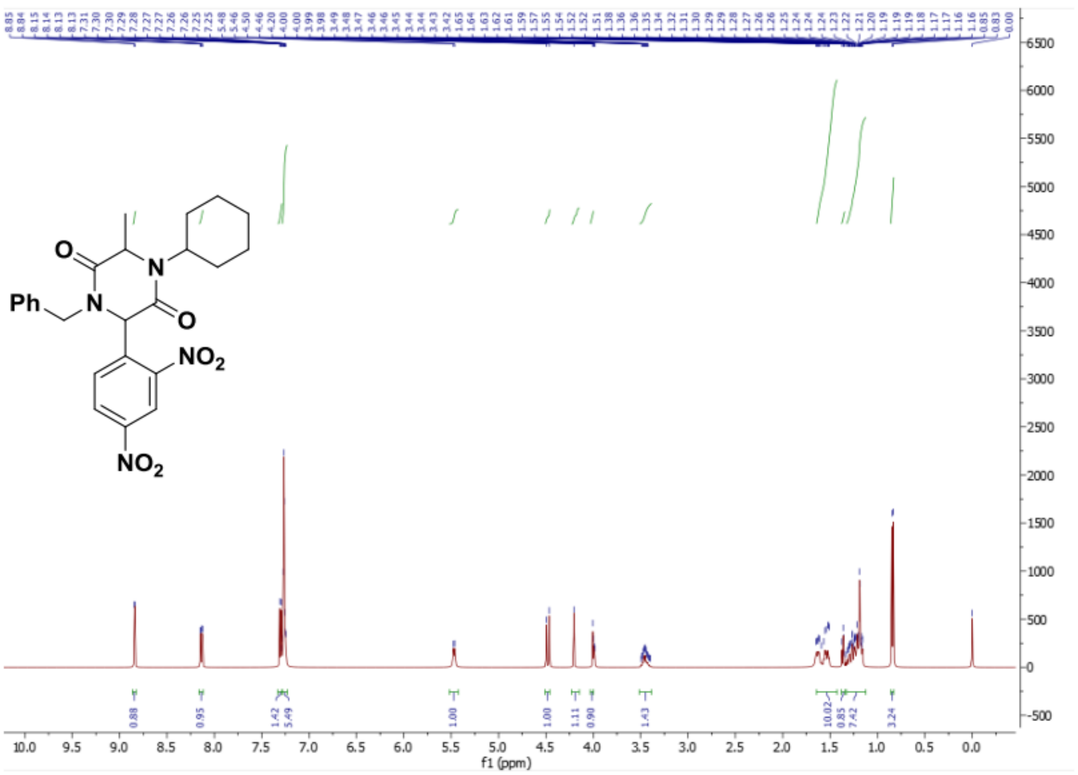

^{13}C NMR:

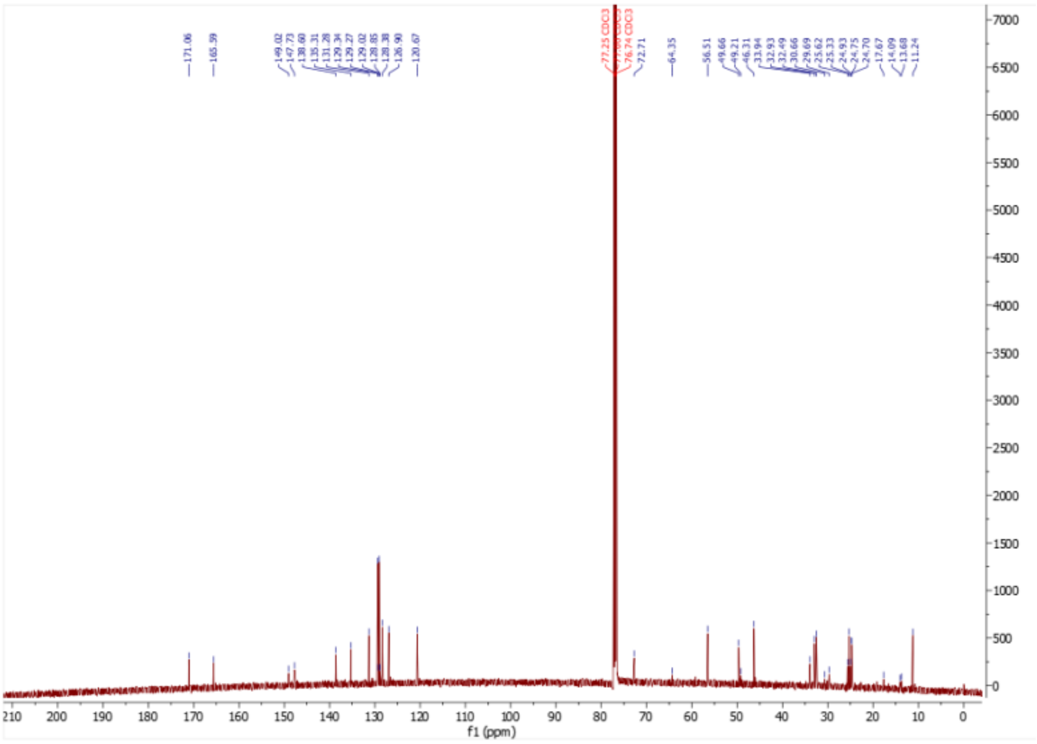

Compound **VIg:**

^{1}H NMR:

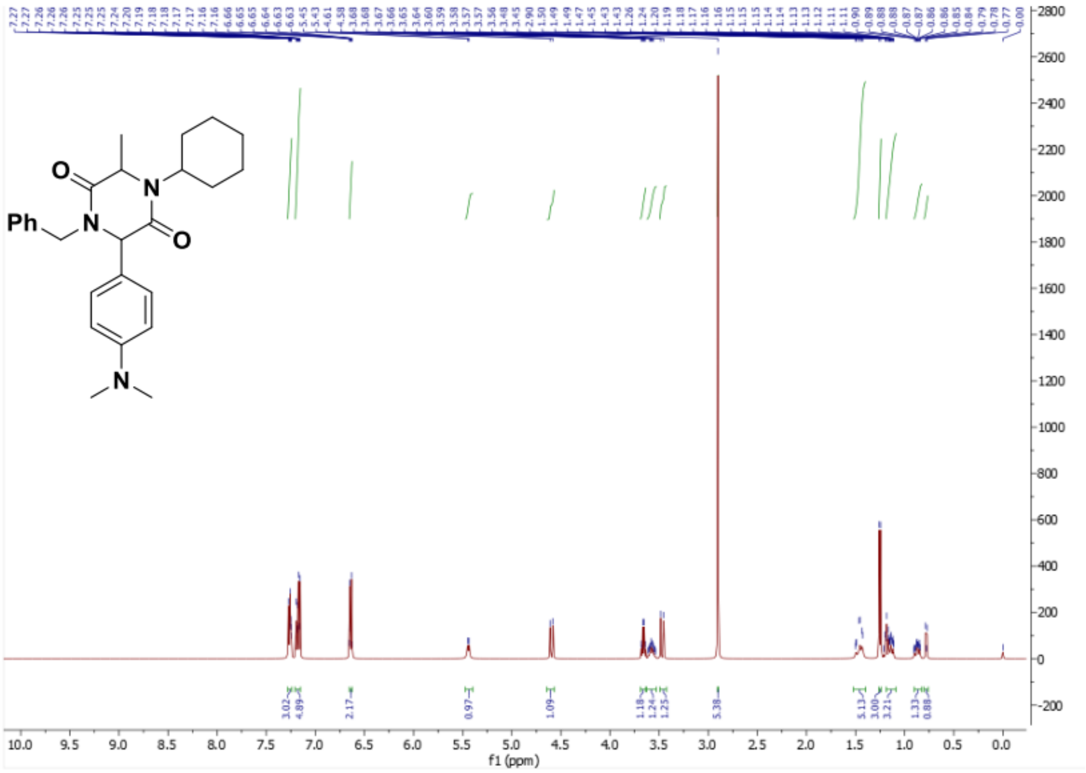

^{13}C NMR:

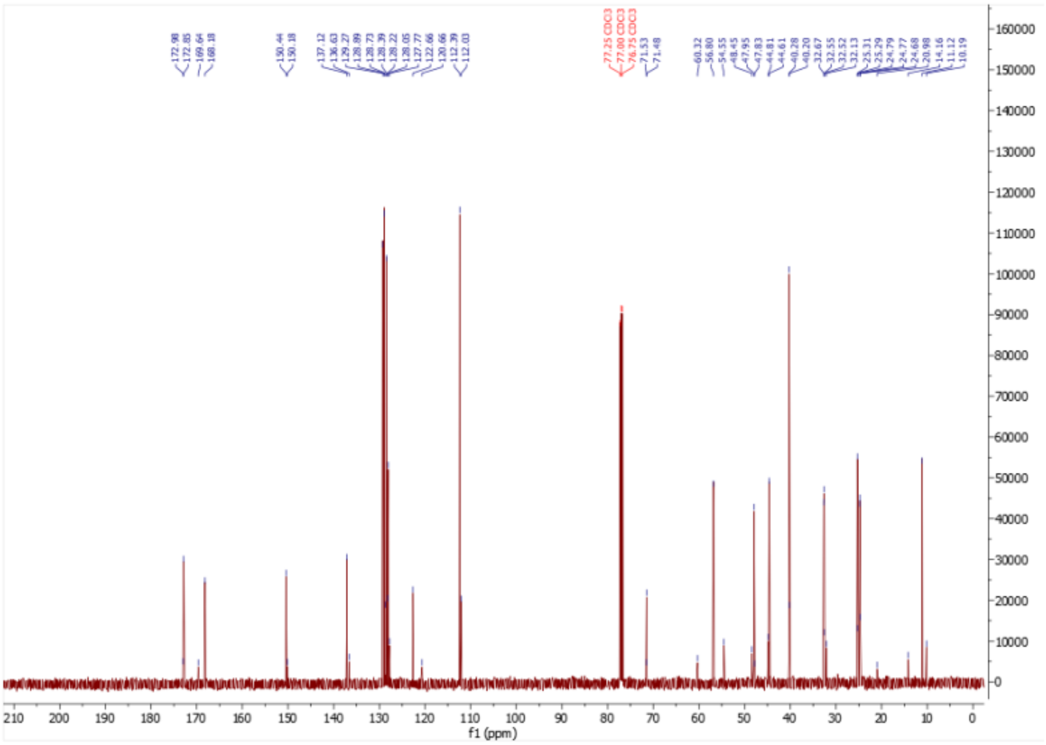

Compound **VIh:**

^{1}H NMR:

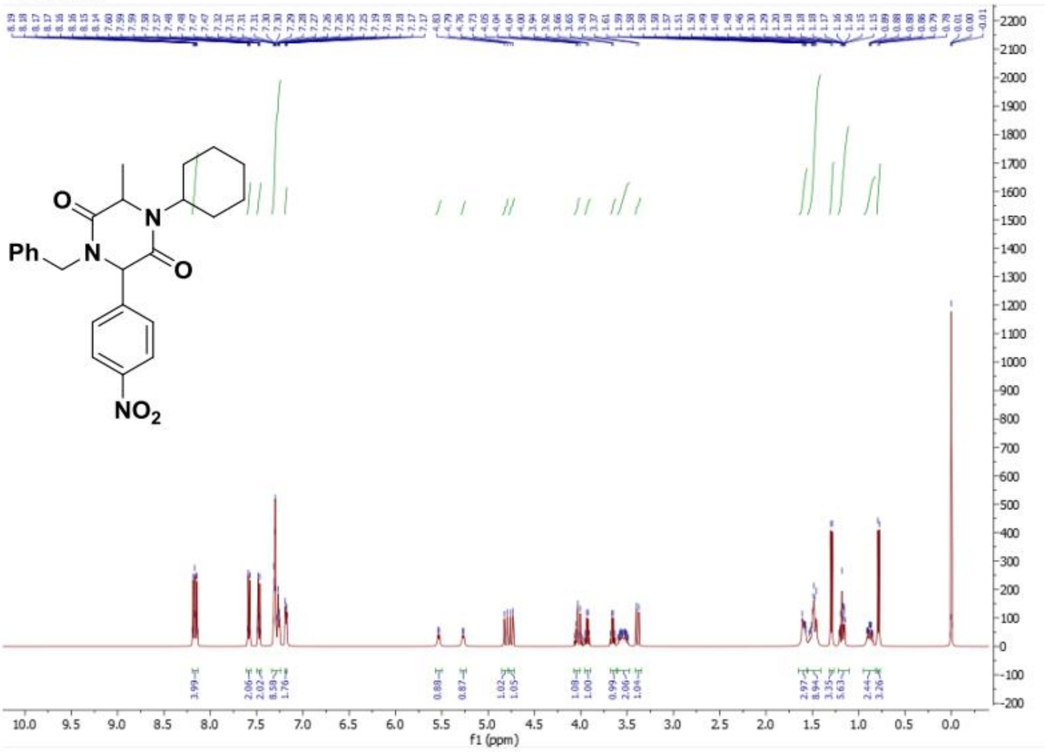

^{13}C NMR:

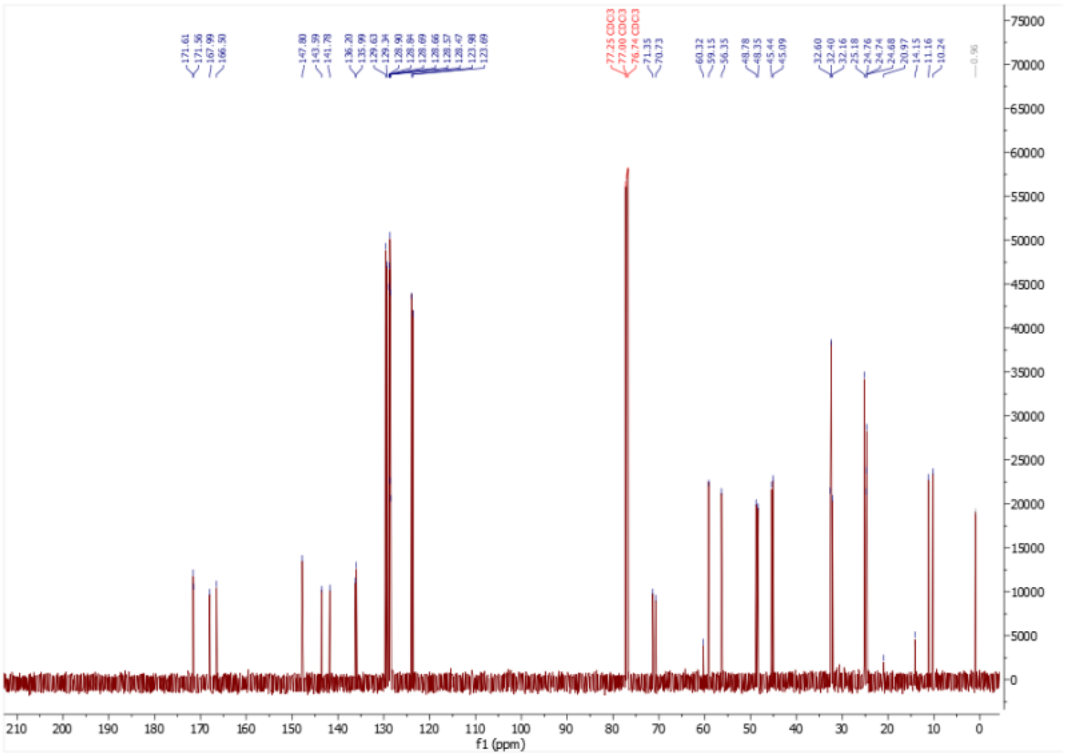

Figure S5. NMR spectra of synthesised compound **Va-Vh & VIa-VIh**
